# A Dataset for Constructing the Network Pharmacology of Overactive Bladder and Its Application to Reveal the Potential Therapeutic Targets of Rhynchophylline

**DOI:** 10.3390/ph17101253

**Published:** 2024-09-24

**Authors:** Yan Tie, Jihan Liu, Yushan Wu, Yining Qiang, Ge’Er Cai’Li, Pingxiang Xu, Ming Xue, Liping Xu, Xiaorong Li, Xuelin Zhou

**Affiliations:** 1 Department of Pharmacology, School of Basic Medical Sciences, Capital Medical University, Beijing 100069, China; 122020010233@mail.ccmu.edu.cn (Y.T.); liujihan@stu.pku.edu.cn (J.L.); wuyushan@mail.ccmu.edu.cn (Y.W.); qiangyn@ydcmdei.org.cn (Y.Q.); clge1@139.com (G.C.); xupingxiang66@ccmu.edu.cn (P.X.); xuem@ccmu.edu.cn (M.X.); 2 School of Chinese Medicine, Capital Medical University, Beijing 100069, China; xulp@ccmu.edu.cn; 3 Department of Pharmacology, School of Basic Medical Sciences, Peking University, Beijing 100191, China

**Keywords:** overactive bladder, network pharmacology, rhynchophylline, target identification

## Abstract

**Objectives**: Network pharmacology is essential for understanding the multi-target and multi-pathway therapeutic mechanisms of traditional Chinese medicine. This study aims to evaluate the influence of database quality on target identification and to explore the therapeutic potential of rhynchophylline (Rhy) in treating overactive bladder (OAB). **Methods**: An OAB dataset was constructed through extensive literature screening. Using this dataset, we applied network pharmacology to predict potential targets for Rhy, which is known for its therapeutic effects but lacks a well-defined target profile. Predicted targets were validated through in vitro experiments, including DARTS and CETSA. **Results**: Our analysis identified Rhy as a potential modulator of the M3 receptor and TRPM8 channel in the treatment of OAB. Validation experiments confirmed the interaction between Rhy and these targets. Additionally, the GeneCards database predicted other targets that are not directly linked to OAB, corroborated by the literature. **Conclusions**: We established a more accurate and comprehensive dataset of OAB targets, enhancing the reliability of target identification for drug treatments. This study underscores the importance of database quality in network pharmacology and contributes to the potential therapeutic strategies for OAB.

## 1. Introduction

Overactive bladder (OAB) is a disease with symptoms including urinary frequency, urgency, and increased nocturia due to abnormal function of the detrusor [1]. A study in the United States showed that 16.6% of adults have OAB symptoms, with a prevalence of 16.9% in women and 16.0% in men [2]. An Asian study indicated that the overall prevalence of OAB was 20.8% (22.1% in women and 19.5% in men) and increased significantly with age, from 10.8% for 40–44 years to 27.9% for >60 years [3]. OAB deeply affects the quality of patients’ lives, causing social dysfunction, depression, etc. The recommended treatment in the revised guidelines jointly written by the American Urological Association and the Society of Urodynamics is bladder training or drug treatments including using an M3 receptor (CHRM3) blocker [4]. However, such M3 receptor antagonists cause common side effects such as constipation and dry mouth, and patients who are not sensitive to M3 receptor antagonists need to double their medication, which seriously affects their quality of life [5]. In recent years, there have also been studies on sacral nerve electrical stimulation, neuromodulation, and surgery for OAB treatment, but the efficacy is not significant [6,7,8]. Therefore, there is a need to develop new drugs for OAB treatment that are safe, effective, and economical.

Uncariae Ramulus Cum Uncis (“Gou-Teng” in Chinese) is the dried hook-bearing stems of *Uncaria rhynchophylla* (Miq.) Miq. ex Havil, *Unvaria macrophylla* Wall., *Uncaria hirsuta* Havil., or *Uncria sessilifructus* Roxb. Uncariae Ramulus Cum Uncis has played a crucial role in traditional Chinese medicine, as evidenced by ancient books like the *Shennong Bencao Jing*. These books emphasize its use in balancing bodily functions and addressing various ailments, including hypertension and epilepsy, reflecting its long-standing importance in treating diverse health conditions [9,10]. At present, while comprehensive toxicity studies on Uncariae Ramulus Cum Uncis are lacking, several studies have provided pharmacokinetic information on its active alkaloids, including rhynchophylline (Rhy) [11], to facilitate researchers to better understand the biosafety of Rhy. As a key active compound of Uncariae Ramulus Cum Uncis, Rhy has demonstrated significant pharmacological effects, such as enhancing heart muscle contractility and increasing calcium transient amplitudes in type 2 diabetic mice [12]. Rhy also reduces oxidative stress markers and calcitonin gene-related peptide (CGRP) levels in nitroglycerin-induced migraine models, suggesting its potential for managing migraine symptoms [13]. Particularly notable is Rhy’s effectiveness in reducing urodynamic and urethral electrophysiologic indices associated with OAB, though the exact mechanisms are still being investigated [14,15,16]. Given the involvement of calcium ions and oxidative stress in OAB, Rhy’s actions might influence these pathways [17]. Future research should focus on elucidating Rhy’s mechanisms and exploring its broader therapeutic potential, enhancing its clinical applications and effectiveness in treating a variety of conditions.

**Figure 1:**
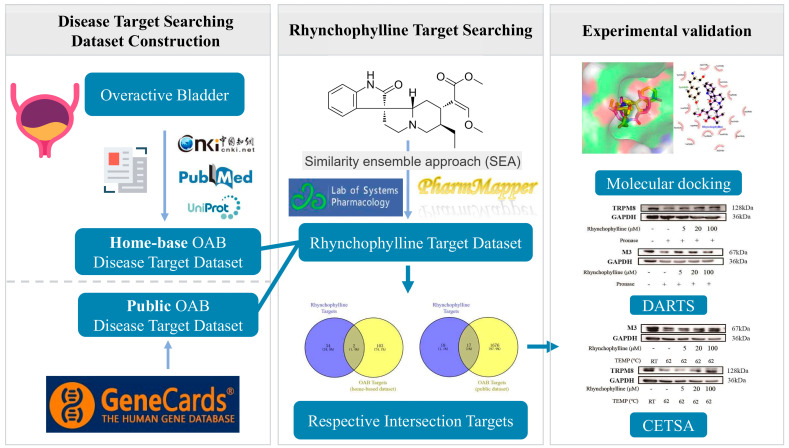
Workflow of the current study.

The network pharmacology approach, as an emerging research method, provides a new research tool for the transformation of traditional Chinese medicine (TCM) from empirical practice to evidence-based medical systems, which can accelerate drug discovery in TCM and improve current drug discovery strategies [18,19]. Relevant target datasets are a prerequisite for conducting network pharmacology. However, the current OAB target datasets are not carefully curated and may even contain some false-positive targets, which poses a major problem for the subsequent analysis. Therefore, the development of OAB-related network pharmacology studies first requires the establishment of relevant and accurate datasets. In this study, we searched for recent publications from the past 20 years to collect evidence-based targets of OAB and set up a dataset. Further, network pharmacology was performed to compare the efficacy and accuracy of the home-based dataset and public database, and we used in vitro experiments to confirm the targets of Rhy (Figure 1). Our current results offer a foundational reference for future experimental studies and clinical applications of Uncariae Ramulus Cum Uncis and its active compound Rhy.

## 2. Results

### 2.1. The Creation of the OAB Disease Target Dataset

This study collected studies from 2006 to 2024 in the literature to provide a more comprehensive overview of a total of 105 drug targets for OAB from 47 published articles. The dataset (version 2.0) has been uploaded to the Population Health Data Archive. Investigators can access the UniProt [20] interface of the relevant target directly from this dataset, which is helpful to further understand the specific information of the target and provide a reference basis for subsequent experimental studies and clinical use. We also searched the GeneCards database [21] for totaling 1693 disease targets of OAB, and their gene symbols, protein names, and relevance scores can be found in Supplemental Table S1.

### 2.2. KEGG and GO Enrichment Results

**Figure 2:**
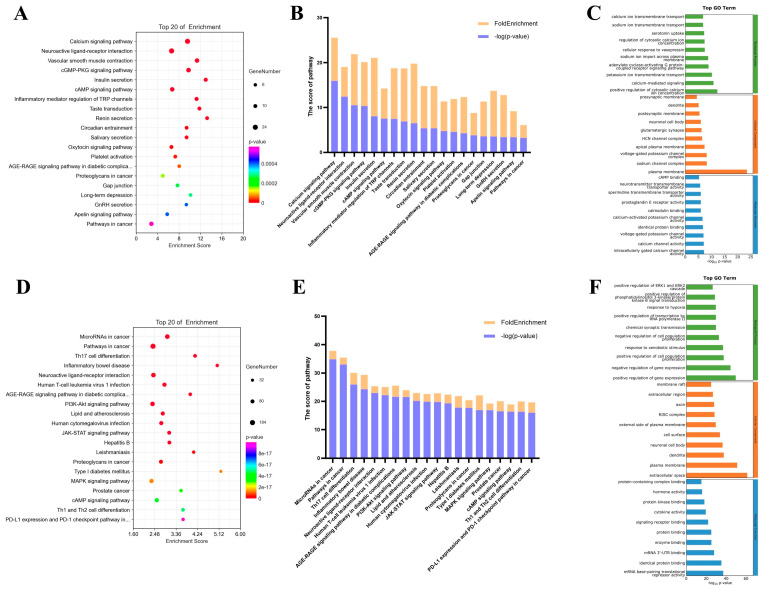
KEGG and GO enrichment results. The KEGG and GO enrichment analysis based on self-built dataset (**A**–**C**) and the GeneCards database (**D**–**F**). (**A**,**B**), the KEGG enrichment based on self-built database; (**C**), the GO enrichment based on self-built dataset; (**D**,**E**), the KEGG enrichment based on GeneCards database; (**F**), the GO enrichment based on GeneCards database.

To demonstrate the differences between these two datasets, we conducted Kyoto Encyclopedia of Genes and Genomes (KEGG) [22,23] and Gene Ontology (GO) [23] analyses on the OAB targets obtained from each. As shown in Figure 2, our database yielded more accurate OAB-related targets, resulting in signaling pathways such as the calcium signaling pathway, neuroactive ligand–receptor interaction, smooth muscle contraction, cyclic guanosine monophosphate–protein kinase G (cGMP–PKG) signaling pathway, cyclic adenosine monophosphate (cAMP) signaling pathway, inflammatory mediator regulation of transient receptor potential (TRP) pathway, etc.; both analyses are known to be closely implicated in OAB according to existing literature being enriched. In contrast, GeneCards provided numerous targets not directly linked to OAB, leading to the enrichment of pathways, such as microRNAs in cancer and pathways in cancer, that are not relevant to OAB.

### 2.3. Predicted Toxicity of Rhynchophylline

The ProTox 3.0 website (https://tox.charite.de/protox3; accessed on 1 September 2024) can predict 50% of lethal dose (LD_50_) value in mg/kg body weight for the acute rodent oral toxicity in mouse or rat experiments [24]. Rhy’s toxicity prediction was carried out based on this method. Toxicity classes are defined according to the globally harmonized system of classification of labeling of chemicals (GHS), predicting various toxicity endpoints through molecular similarity. According to the prediction of LD_50_ value, the toxicity level of compounds is divided into six categories, among which Rhy belongs to the fourth category [harmful if swallowed (300 mg/kg < LD_50_ ≤ 2000 mg/kg)], with an LD_50_ value of 325 mg/kg. Meanwhile, the predicted LD_50_ value of acetaminophen is 338 mg/kg, which is very close to the known hepatotoxic dose for humans. Thus, Rhy was predicted with a toxicity as low as acetaminophen, a drug with high safety.

### 2.4. Rhynchophylline Targets and Intersecting Targets

**Figure 3:**
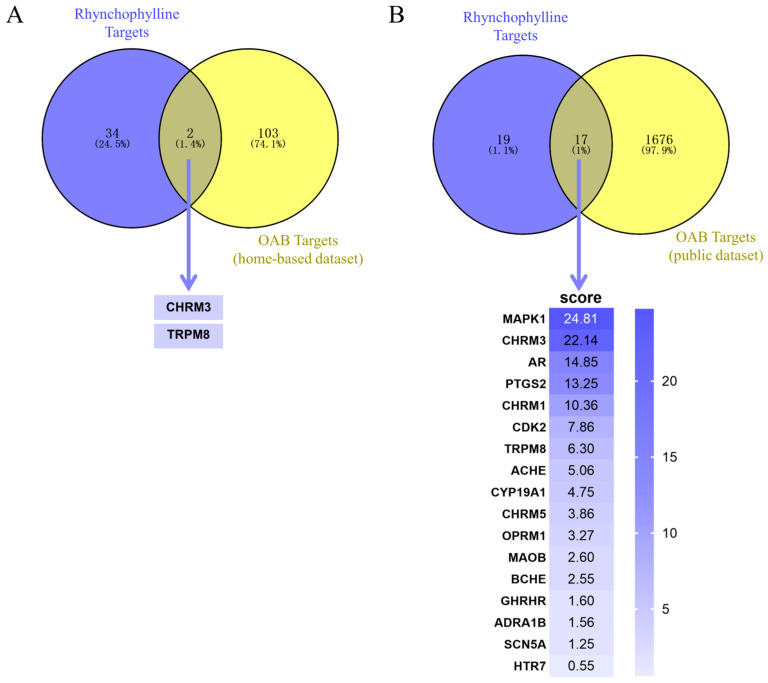
Drug–disease intersecting targets. (**A**) Intersecting targets collected from the self-built dataset. (**B**) Intersecting targets collected from public databases.

A total of 36 drug targets in Rhy were screened through the Traditional Chinese Medicine Systems Pharmacology Database and Analysis Platform (TCMSP, https://old.tcmsp-e.com/tcmsp.php; accessed on 29 May 2024) [25], PharmMapper (http://lilab-ecust.cn/pharmmapper; accessed on 29 May 2024) [26], and Similarity Ensemble Approach Database (SEA, https://sea.bkslab.org; accessed on 29 May 2024) [27] (Supplemental Figure S1). By Venn diagram analysis, there are two intersecting targets, muscarinic acetylcholine receptor M3 (CHRM3) and transient receptor potential cation channel subfamily M member 8 (TRPM8), in our dataset and Rhy’s targets (Figure 3A); meanwhile, the GeneCards database has 17 targets (Figure 3B), whose gene names and scores are shown as well. However, the intersection targets CHRM3 and TRPM8 obtained from our dataset do not score well in the public dataset. To verify our findings, further in silico and in vitro experiments were performed.

### 2.5. Resutls for Molecular Docking Analysis

**Figure 4:**
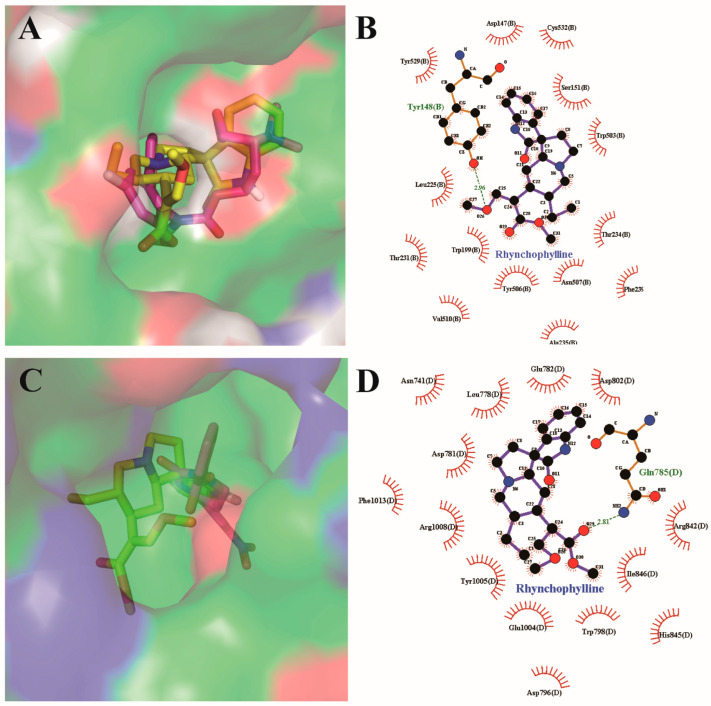
Molecular docking analysis reveals the best binding positions of the Rhy (sticks in yellow) with the lowest binding free energy in the ligand-binding cavity of CHRM3 (PDB ID 5ZHP) and TRMP8 (PDB ID 7WRC). (**A**) The three-dimensional diagram illustrates the interactions of Rhy (yellow sticks) with CHRM3 at the same binding position of crystallized 9EC (purple sticks). (**B**) The two-dimensional diagram indicates the interactions of Rhy with the amino acid residues in the binding pocket of CHRM3. (**C**) The three-dimensional diagram illustrates the interactions of Rhy (yellow sticks) with TRMP8 at the same binding position of crystallized icilin (purple sticks). (**D**) The two-dimensional diagram indicates the interactions of Rhy with the amino acid residues in the binding pocket of TRMP8. Green lines show H-bonding with different distances between ligands and specific amino acid residues. The spoked arcs indicate amino acid residues providing non-bonded interactions with the ligand.

From our previous study [28], the 3D conformation of the docked 9EC in the ligand-binding cavity of CHRM3 (Protein Data Bank, www.rcsb.org, PDB ID 5ZHP, https://doi.org/10.2210/pdb5ZHP/pdb; accessed on 5 July 2024) was with binding free energy of −11.5 kcal/mol, indicating high binding affinity to CHRM3. Meanwhile, Rhy bound to this cavity with −8.2 kcal/mol at the same binding site (Figure 4A). Two-dimensional conformation analysis showed that Rhy strongly interacted with Tyr148 via H-bonding at a distance of 2.96 Å and obtained hydrophobic interactions with Ser151, Trp503, Trp199, Tyr506, etc. (Figure 4B). 

For the docking simulation to the ligand-binding site of TRMP8, the 3D conformation of the redocked icilin in the ligand-binding cavity of TRMP8 (PDB ID 7WRC, PDB DOI: https://doi.org/10.2210/pdb7WRC/pdb; accessed on 5 July 2024) [29] was with binding free energy of −10.2 kcal/mol at the same binding site of crystalized icilin. Meanwhile, Rhy bound to this cavity with −8.1 kcal/mol at the same binding site (Figure 4C). Two-dimensional conformation analysis showed that Rhy strongly interacted with Gln785 via H-bonding at a distance of 2.81 Å and obtained hydrophobic interactions with Glu782, Leu778, Tyr1005, Ile846, etc. (Figure 4D). The rigid docking results supported that Rhy can bind to CHRM3 and TRMP8, respectively, which is similar to the findings from network pharmacology with the self-built dataset. In the future, flexible docking analysis should be performed to obtain a much better result for assessing the best binding performance of Rhy.

### 2.6. Verification of the Binding of Rhy to CHRM3 and TRPM8

**Figure 5:**
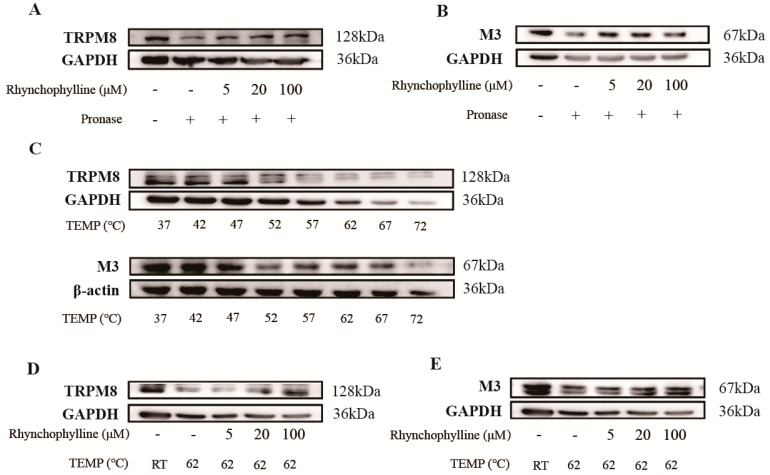
Identification of TRPM8 and M3 as direct targets of Rhy. (**A**) The DARTS–Western blotting experiment was used to verify that TRPM8 significantly increased in tissue lysate with Rhy. (**B**) The DARTS–Western blotting experiment was used to verify that M3 significantly increased in tissue lysate with Rhy. (**C**) CETSA temperature gradient experiment. (**D**) The CETSA experiment was used to verify that the degradation degree of TRPM8 with Rhy was lower than that of the HT group. (**E**) The CETSA experiment was used to verify that the degradation degree of M3 with Rhy was lower than that of the HT group.

We used the drug affinity responsive target stability (DARTS) and cellular thermal shift assay (CETSA) experiments to verify the binding effect of Rhy to the TRPM8 channel and CHRM3. The experimental results of DARTS experiments are shown in Figure 5. After incubating Rhy (5~100 μM) with bladder protein lysate for 1 h, the protein bands of TRPM8 and CHRM3 in the Rhy treatment gradually deepened with increasing concentrations (Figure 5A,B). In addition, we also validated them using DARTS experiments. According to the temperature gradient results (Figure 5C), the degradation degree of TRPM8 and CHRM3 was observed at 62 °C. At the same temperature (62 °C), the degradation degree of TRPM8 protein and CHRM3 in high-temperature control (HC) samples was significantly higher than that of HC-Rhy (from 5 μM) (Figure 5D,E). Consistent with the predicted results, Rhy stabilized the TRPM8 channels and CHRM3 in the tissue lysate against enzymatic degradation and heat degradation. Therefore, these data confirmed that Rhy has a direct binding effect on the TRPM8 channel and CHRM3.

## 3. Discussion

Network pharmacology is an innovative field within pharmacology that integrates systems biology and network analysis to create intricate and systematic networks of “disease–phenotype–gene–compound” interactions. This methodology facilitates a deeper understanding of the complex mechanisms underlying the multifaceted actions of drugs [30,31]. The holistic philosophy of TCM has much in common with the key ideas of network pharmacology and meets the requirements of overcoming complex diseases or mechanisms in a systematic way [32,33]. Network pharmacology as a promising research method holds considerable promise for advancing drug discovery and elucidating mechanistic pathways, especially for TCM/herbal extracts with complicated components.

The prerequisite for the application of network pharmacology is the construction of a “drug–component–target–disease” network [30,34]. The collection of disease targets has always been a problem for researchers, and although there are various online databases that summarize disease targets, their results always differ [35,36]. For example, in this study, the numbers of OAB disease targets obtained from different databases are inconsistent, GeneCards database can provide 1694 targets, DISGENET (https://disgenet.com; accessed on 29 May 2024) [37] can provide 201 targets, Pharmacogenetics and Pharmacogenomics Knowledge Base (PHARMGKB, www.pharmgkb.org; accessed on 29 May 2024) [38] can provide 995 targets, and the human disease database Online Mendelian Inheritance in Man (OMIM, www.omim.org; accessed on 29 May 2024) [39] does not contain information on the disease targets of OAB. Our study first established a dataset of therapeutic targets related to OAB through an extensive review of 47 published articles and compared it to the public datasets. Based on our dataset and the network pharmacology research method, combined with our previous pharmacodynamic research, this study tried to analyze the pharmacological mechanisms of Rhy to prove the reliability of our self-built dataset, and also provide a basis for its potential clinical use.

GeneCards is a compendium of human genes that brings together data from more than 150 databases, enabling researchers to efficiently browse and correlate a wide range of human genes, diseases, variants, proteins, cells, and biological pathways [21]. For databases as large as GeneCards, automated data mining can quickly extract and annotate large amounts of data from multiple sources and large amounts of data [40,41,42]. However, automated mining methods can lead to false-positive or false-negative annotations, which can lead to improper disease targets prediction [43,44]. Besides, different algorithms applied to different databases can cause discrepancies and lead to errors [45]. In order to avoid the above-mentioned inaccuracy, in this study, we collected the literature through different literature search platforms and obtained 112 OAB disease targets after integration and merging protein targets. As all the targets were collected manually, the accuracy of the data was guaranteed. To validate this, we selected the Rhy for the validation of network pharmacology using our self-built database compared with public databases.

By taking intersections of disease target databases and Rhy targets, we found that the two intersecting targets, CHRM3 and TRPM8, were scored well in the self-built dataset, but they were not scored well in the public dataset. GeneCards databases were using Elasticsearch v.7.11 (https://www.elastic.co/elasticsearch; accessed on 29 May 2024) to score the targets. They use a Boolean model to find matching documents and a formula called the utility scoring function to calculate the correlation [46,47,48]. They mainly use term frequency, inverse document frequency, field-length norm, and vector space model to formulate the formula to calculate and rank the relevance of each target [49,50,51]. This presents some problems when counting disease targets, such as inconsistent disease names or different disease sub-types, which can affect the score derived from the database [52,53]. Similarly, the writing habits of different authors may cause the term frequency to vary, resulting in target scoring errors [54].

In our study, CHRM3 received a score of 22.14 and TRPM8 received a score of 6.3 in the public dataset, ranking second and seventh, respectively. Subsequently, DARTS and CETSA experiments were applied to confirm the binding of Rhy to CHRM3 and TRPM8. CHRM3 has been shown to play an important role in the development of OAB [55]. Detrusor contraction can be triggered by an agonist of the muscarinic receptor, and M receptor antagonists can inhibit detrusor over-contraction, improve bladder sensory function, and inhibit unstable contraction of the detrusor possibly by antagonizing the M receptor; therefore, they are widely used for the treatment of OAB [56,57]. Among them, CHRM3 antagonists, such as darifenacin, have become the first-line drugs for OAB [58,59]. Meanwhile, TRPM8 may be associated with pathophysiologic overactivity of bladder-afferent nerves [60]. One study reported an increase in TRPM8-positive cells and mRNA in both the bladder and dorsal root ganglia of rats after bladder outlet obstruction [61]. Similarly, the combination of the TRPM8 antagonist KPR-5714 with a β (3)-adrenergic receptor agonist or an anticholinergic drug enhances the efficacy of bladder dysfunction in OAB rats [62].

The top-ranked target MAPK1 (mitogen-activated protein kinase 1) in the public dataset has been less frequently reported to be associated with OAB. MAPK1, also known as extracellular signal-regulated kinases (ERKs), serves as an integration site for a variety of biochemical signals and is involved in a variety of cellular processes such as proliferation, differentiation, transcriptional regulation, and development [63,64,65]. It is hypothesized that phosphorylation of ERKs affects actin expression, thereby enhancing smooth muscle contraction, and ERKs are also the targets against cystitis [66,67,68,69]. However, smooth muscle contraction involves the binding of myosin and actin [70,71]. Many animal models of OAB exhibit insignificant changes in the protein expression of actin in the bladder, while changes in the phosphorylation of the myosin light chain are common; moreover, myosin inhibitors can relax the detrusor in vitro and in vivo [72,73,74]. Thus, regulating actin as a target effector protein may not work well for most OAB bladders. This suggests that MAPK1 may not be a good target for OAB. Therefore, both targets predicted by our dataset were shown to be associated with OAB; however, the public dataset did not have this precision.

In summary, the summarization of disease targets is particularly essential for the subsequent efficacy and mechanism studies in network pharmacology, which is important for future experiment validation. Although existing disease target databases, such as GeneCards and OMIM, are well known and extensively used, there are some limitations for studies due to the large variations and the emergence of false positives. The researchers using these databases have to carefully validate their predicted results. If they are overly dependent on these invalidated predictions to explore the therapeutic mechanisms of herbal extracts or compounds, they may obtain false-positive results. Our self-constructed database obtained by organizing the literature carefully ensured the accuracy of the prediction, and the predicted targets were also verified by experiments. Moreover, in the future, we will further update our dataset to keep up with the new literature irregularly.

## 4. Materials and Methods

### 4.1. Identification of Potential Targets of OAB

The dataset for OAB was obtained by finding information on therapeutic targets related to OAB in PubMed (pubmed.ncbi.nlm.nih.gov; accessed since 1 November 2020) and China National Knowledge Infrastructure (www.cnki.net; accessed since 1 November 2020) from 47 published articles. The screened target protein names were then converted to gene names by using the UniProt database (https://www.uniprot.org; accessed since 1 November 2020), and duplicate therapeutic targets were merged, while target information for which gene names could not be found was also removed. The resulting dataset contains the UniProt links corresponding to the targets and the corresponding references. We also obtained OAB disease targets from the GeneCards database (https://www.genecards.org; accessed on 29 May 2024) for comparison. Besides, KEGG and GO enrichment analyses were conducted on the targets obtained from both databases to illustrate the distinctions between them.

### 4.2. Prediction of Toxicity of Rhynchophylline

To ensure the biological safety of Rhy, the ProTox v.3.0 database was used (https://tox.charite.de/protox3; accessed on 1 September 2024) to predict their acute toxicity [24]. A control drug acetaminophen with high clinical safety was also tested. The SMILE data were collected from Pubchem (Acetaminophen canonical SMILE: CC(=O)NC1=CC=C(C=C1)O; Rhy canonical SMILE: CCC1CN2CCC3(C2CC1C(=COC)C(=O)OC)C4=CC=CC=C4NC3=O). As a result, the LD_50_ values of these compounds were collected for comparison.

### 4.3. Collection and Screening of Rhynchophylline’s Targets

The data of drug targets contained in Rhy were mined by retrieving from the Traditional Chinese Medicine Systems Pharmacology Database and Analysis Platform (https://old.tcmsp-e.com/tcmsp.php; accessed on 29 May 2024), PharmMapper (http://lilab-ecust.cn/pharmmapper; accessed on 29 May 2024), and SEA Database (https://sea.bkslab.org; accessed on 29 May 2024). The targets obtained from each database were then merged to construct a dataset of Rhy’s drug targets.

### 4.4. Acquisition of Disease and Drug Intersection Targets

In order to compare the correctness of the GeneCards database and our self-built database, we obtained the intersecting targets of these two databases with Rhy by VENNY v.2.1 (https://bioinfogp.cnb.csic.es/tools/venny; accessed on 29 May 2024).

### 4.5. Molecular Docking Analysis

To facilitate a better understanding of the binding of Rhy to the intersection-targeting proteins, molecular docking analysis was used for modeling with AutoDock Vina v.1.0.2 set by default parameters [75]. Rigid docking was used to obtain an unbiased result without additional interference. As a control result, the binding positions of re-docked ligands from the crystallized protein structures should be close to their original ones. The protein crystal structures were collected from the Protein Data Bank. If the protein structures were not listed, AlphaFold [76] was used to generate the 3D structures of the target proteins. The molecular docking conformation was further selected by choosing the binding mode of the compounds for the protein target with the lowest binding affinity. Simulation results were illustrated by PyMOL Molecular Graphics System [21] v.1.3 (Schrödinger, LLC, New York, NY, USA) and LigPlot^+^ v.2.2 [77] (https://www.ebi.ac.uk/thornton-srv/software/LigPlus; accessed on 4 July 2024), respectively.

### 4.6. Chemicals 

The Rhy (CAS No. 76-66-4; purity ≥ 98%) used in our study was provided by Shanghai Macklin Biochemical Technology Co., Ltd. (Cat No. R817208, Shanghai, China).

### 4.7. Animals

Male Sprague-Dawley (SD) rats (200 ± 20 g) were provided by Beijing Vital Laboratory Animal Technology Co., Ltd. (SCXK 2021-0006, Beijing, China) and the experimental procedures were approved by the Ethics Review Committee for Animal Experimentation of Capital Medical University (Ethical Approval Number AEEI-2022-067). Briefly, the SD rats were sacrificed with CO_2_ and the bladder tissue was collected immediately and frozen with liquid nitrogen then stored at −80 °C. Next, when used, the tissues were homogenized with lysis buffer containing protein inhibitor cocktail.

### 4.8. Drug Affinity Responsive Target Stabilization Assay (DARTS)

To verify the predicted results, the drug affinity responsive target stabilization assay (DARTS) and cellular thermal shift assay (CETSA) were applied, respectively. Lysis buffer was prepared using mammalian active protein extraction reagent (M-Per, Beyotime, P0013M-100 mL) and protein inhibitor cocktail (Beyotime, P1048-1 mL, 50×) at a ratio of 50:1. Pre-cooled lysis buffer (10 μL/mg) was added to bladder tissue, then the tissue was homogenized with a homogenizer and centrifuged to collect the supernatant. Protein concentration was determined according to the instructions of the BCA reagent kit (Omni-Easy™ Ready-to-use BCA Protein Assay Kit, Epizyme Biotech, Shanghai, China). TNC solution (10×; 0.5 M Tris·HCl, 0.5 M NaCl, 0.1 M CaCl_2_) was added to the supernatant of the protein solution at a ratio of 1:9 to achieve a protein concentration of 5 mg/mL. The protein solution was incubated at room temperature with Rhy for 1 h. Then, the protein solution was digested with pronase at a ratio of 1:400 for 30 min. Finally, the 5× loading buffer was added and boiled for 10 min. All samples were validated through Western blotting experiments with respective antibodies.

### 4.9. Cellular Thermal Shift Assay (CETSA)

After adding tissue lysis solution, the bladder tissue was homogenized and centrifuged, and then the supernatant protein solution was subjected to quantification by using the BCA kit. Part of the tissue lysis solution was sequentially heated at a range of temperatures (37~72 °C at intervals of 5 °C) for 5 min as a pre-experiment, and 62 °C was determined as the final degradation temperature. Then, the remaining tissue lysis solution was divided into normal atmospheric temperature control (NC) samples, high-temperature control (HC) samples and high-temperature-Rhy (HC-Rhy) treatment samples. After incubating with Rhy for 1 h, both the HC and HC-Rhy were all heated at 62 °C for 5 min. Then, all samples were centrifuged at 12,000× *g* for 10 min at 4 °C, added to the loading buffer, and boiled for 10 min. All samples were validated through Western blotting experiments with respective antibodies.

### 4.10. Western Blotting

The samples prepared above were quantified by the BCA protein assay and denatured at 100 °C for 5 min. Then, they were separated by SDS-PAGE and transferred to the PVDF membrane. After being blocked by 5% non-fat milk, the membranes were incubated with primary antibodies overnight at 4 °C, including CHRM3 (1:5000, Abcam, Shanghai, China, Cat No. ab87199) and TRPM8 (Affinity, Jiangsu, China, 1:2000, Cat No. DF7966). The membranes were subsequently incubated with appropriate HRP-conjugated secondary antibodies. Finally, the protein blots were visualized and detected by chemiluminescence detection with an enhanced chemiluminescence kit (NcmECL Ultra, Cat No. P10300, New Cell & Molecular Biotech Co., Ltd., Suzhou, China).

## 5. Conclusions

We constructed a new disease dataset about OAB through an extensive literature search, which initially predicted the potential targets of rhynchophylline in the treatment of OAB, and experimental validation confirmed that rhynchophylline binds to two important OAB targets, CHRM3 and TRPM8. A comparative analysis between our self-constructed dataset and public databases revealed that our dataset provided more accurate and reliable predictions. In the future, additional ex vivo experiments including a detrusor muscle strips study and in vivo animal experiments should be conducted to further validate the pharmacological effects and confirm the targets of rhynchophylline in the treatment of OAB.

## Data Availability

The dataset (version 2.0) of therapeutic targets of OAB can be accessed through the following link (https://www.ncmi.cn/phda/dataDetails.do?id=CSTR:A0006.11.Z027Q.202012.79.V1.0; accessed on 16 July 2024).

## Supplementary Materials

The following supporting information can be downloaded at https://www.mdpi.com/article/10.3390/ph17101253/s1: Figure S1. A total of 36 drug targets of rhynchophylline collected from the TCMSP, PharmMapper, and SEA database; Table S1. A total of 1693 disease targets of OAB found in the GeneCards database.
