# Supplementary material for: A Dataset for Constructing the Network Pharmacology of Overactive Bladder and Its Application to Reveal the Potential Therapeutic Targets of Rhynchophylline
Source: Pharmaceuticals (Basel). 2024 Sep 24;17(10):1253. doi: 10.3390/ph17101253 (PMC11510256; doi:10.3390/ph17101253)
Supplement: Supplementary file 1 [file pharmaceuticals-17-01253-s001.zip › pharmaceuticals-3176604-supplementary.pdf]

pharmaceuticals

Article

A Dataset for Constructing Network Pharmacology of Overactive Bladder and Its Application to Reveal the Potential Therapeutic Targets of Rhyncholphylline

Yan Tie[1,2,†], Jihan Liu[1,3,†], Yushan Wu[1,†], Yining Qiang[1], Ge'Er Cai'Li[1], Pingxiang Xu[1], Ming Xue[1], Liping Xu[2], Xiaorong Li[1,*], Xuelin Zhou[1,*]

1 Department of Pharmacology, School of Basic Medical Sciences, Capital Medical University, Beijing 100069 China; 122020010233@mail.ccmu.edu.cn (Y.T.); liujihan@stu.pku.edu.cn (J.L.); wuyushan@mail.ccmu.edu.cn (Y.W.); qiangyn@ydcmdei.org.cn (Y.Q.); clge1@139.com (G.C.); xupingxiang66@ccmu.edu.cn (P.X.); xuem@ccmu.edu.cn (M.X.)
2 School of Chinese Medicine, Capital Medical University, Beijing 100069, China; xulp@ccmu.edu.cn
3 Department of Pharmacology, School of Basic Medical Sciences, Peking University, Beijing 100191, China
* Correspondence: zhouxuelin@ccmu.edu.cn or peterxlzhou@gmail.com (X.Z.); lixiaorong@ccmu.edu.cn (X.L.);
† These authors contributed equally to this work.

Supplementary Materials:

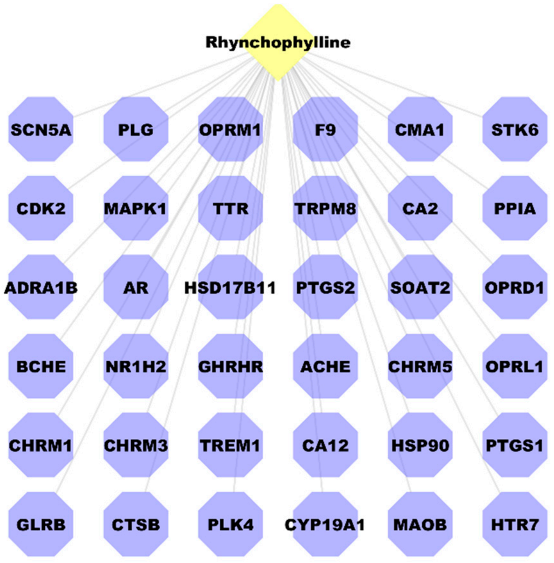

Figure S1. Total 36 drug targets of Rhynchophylline collected from the TCMSP, PharmMapper, and SEA database.

Citation: To be added by editorial staff during production.

Academic Editor: Firstname Lastname

Received: date
Revised: date
Accepted: date
Published: 24 September 2024

Copyright: © 2024 by the authors. Licensee MDPI, Basel, Switzerland. This article is an open access article distributed under the terms and conditions of the Creative Commons Attribution (CC BY) license (https://creativecommons.org/licenses/by/4.0/).

Table S1. Total 1,693 disease targets of OAB searched from the GeneCards database.

No.	Gene Symbol	Description	Relevance score
1	TP53	Tumor Protein P53	77.25287628
2	FGFR3	Fibroblast Growth Factor Receptor 3	63.97473907
3	RB1	RB Transcriptional Corepressor 1	60.22346115
4	HRAS	HRas Proto-Oncogene, GTPase	52.66714859
5	KRAS	KRAS Proto-Oncogene, GTPase	47.56088638
6	CTNNB1	Catenin Beta 1	46.97029495
7	PIK3CA	Phosphatidylinositol-4,5-Bisphosphate 3-Kinase Catalytic Subunit Alpha	46.93548965
8	ERBB2	Erb-B2 Receptor Tyrosine Kinase 2	46.24708176
9	CDKN2A	Cyclin Dependent Kinase Inhibitor 2A	45.86863708
10	TSC1	TSC Complex Subunit 1	43.79502106
11	PTEN	Phosphatase And Tensin Homolog	42.67813492
12	CDKN1A	Cyclin Dependent Kinase Inhibitor 1A	41.81648636
13	EGFR	Epidermal Growth Factor Receptor	41.62625122
14	TERT	Telomerase Reverse Transcriptase	40.70458984
15	H19	H19 Imprinted Maternally Expressed Transcript	37.88514709
16	GAS5	Growth Arrest Specific 5	35.27672577
17	NF1	Neurofibromin 1	34.88474655
18	MEG3	Maternally Expressed 3	33.53116989
19	MALAT1	Metastasis Associated Lung Adenocarcinoma Transcript 1	33.37225342
20	BRAF	B-Raf Proto-Oncogene, Serine/Threonine Kinase	32.74304199
21	MIR145	MicroRNA 145	32.57999802
22	MIR143	MicroRNA 143	32.42773438
23	MIR21	MicroRNA 21	30.2622776
24	AKT1	AKT Serine/Threonine Kinase 1	29.27330971
25	MIR29C	MicroRNA 29c	29.21055603
26	MIR17	MicroRNA 17	29.20332718
27	MIR99A	MicroRNA 99a	29.01646996
28	MIR205	MicroRNA 205	28.66386604
29	XIST	X Inactive Specific Transcript	28.6484375
30	MIR221	MicroRNA 221	28.58753395
31	MIR133B	MicroRNA 133b	28.34265137
32	MIR223	MicroRNA 223	28.30670547
33	MIR195	MicroRNA 195	28.07801437
34	MIR23A	MicroRNA 23a	27.83349419
35	MIR23B	MicroRNA 23b	27.82202721
36	MIR31	MicroRNA 31	27.69780922
37	MIR222	MicroRNA 222	27.67320633
38	MIR30E	MicroRNA 30e	27.21254539
39	MIR185	MicroRNA 185	26.71201324
40	MIR10B	MicroRNA 10b	26.41508293
41	MIR103A1	MicroRNA 103a-1	26.34848595
42	MIR127	MicroRNA 127	26.3395977
43	MTOR	Mechanistic Target Of Rapamycin Kinase	25.44851303
44	CREBBP	CREB Binding Protein	25.09002304

45	MAPK1	Mitogen-Activated Protein Kinase 1	24.81913567
46	NFE2L2	NFE2 Like BZIP Transcription Factor 2	24.56549835
47	SNHG5	Small Nucleolar RNA Host Gene 5	24.42776871
48	CHRNA3	Cholinergic Receptor Nicotinic Alpha 3 Subunit	24.42388344
49	HNF1A-AS1	HNF1A Antisense RNA 1	24.39972878
50	NORAD	Non-Coding RNA Activated By DNA Damage	23.64861488
51	LRIG2	Leucine Rich Repeats And Immunoglobulin Like Domains 2	23.46240807
52	CDH1	Cadherin 1	22.24908829
53	MAP2K1	Mitogen-Activated Protein Kinase Kinase 1	22.15703392
54	CHRM3	Cholinergic Receptor Muscarinic 3	22.14151955
55	ISL1	ISL LIM Homeobox 1	21.90647125
56	NGF	Nerve Growth Factor	20.99368858
57	GSTM1	Glutathione S-Transferase Mu 1	20.61676598
58	UPK2	Uroplakin 2	20.42460823
59	NAT2	N-Acetyltransferase 2	20.22233582
60	UPK3A	Uroplakin 3A	19.94803238
61	HPSE2	Heparanase 2 (Inactive)	19.6267128
62	CCND1	Cyclin D1	19.05813408
63	CDKN1B	Cyclin Dependent Kinase Inhibitor 1B	18.76374435
64	TNF	Tumor Necrosis Factor	18.51833534
65	P2RX3	Purinergic Receptor P2X 3	18.15187263
66	GSTT1	Glutathione S-Transferase Theta 1	18.10890388
67	LMOD1	Leiomodin 1	17.36113739
68	TRPV1	Transient Receptor Potential Cation Channel Subfamily V Member 1	17.12408066
69	IL6	Interleukin 6	16.84867477
70	VEGFA	Vascular Endothelial Growth Factor A	16.64421463
71	HBEGF	Heparin Binding EGF Like Growth Factor	16.60595131
72	TGFB1	Transforming Growth Factor Beta 1	15.94863796
73	GJA1	Gap Junction Protein Alpha 1	15.77251816
74	ADRB3	Adrenoceptor Beta 3	15.60766411
75	MYC	MYC Proto-Oncogene, BHLH Transcription Factor	15.57951641
76	NAT1	N-Acetyltransferase 1	15.5290184
77	GSTP1	Glutathione S-Transferase Pi 1	15.36805344
78	EGF	Epidermal Growth Factor	15.29355049
79	UPK1A	Uroplakin 1A	15.25492477
80	UPK1B	Uroplakin 1B	15.13848019
81	MMP1	Matrix Metallopeptidase 1	15.04410362
82	MMP9	Matrix Metallopeptidase 9	14.97369385
83	AR	Androgen Receptor	14.8502264
84	RHEB	Ras Homolog, MTORC1 Binding	14.84452057
85	CXCL8	C-X-C Motif Chemokine Ligand 8	14.78918457
86	MKI67	Marker Of Proliferation Ki-67	14.65207386
87	PDCD1	Programmed Cell Death 1	14.59262085
88	MMP2	Matrix Metallopeptidase 2	14.50840473

89	FGFR2	Fibroblast Growth Factor Receptor 2	14.33659172
90	CCL2	C-C Motif Chemokine Ligand 2	14.29826736
91	VIM	Vimentin	14.11194324
92	CHRM2	Cholinergic Receptor Muscarinic 2	13.9348774
93	SRC	SRC Proto-Oncogene, Non-Receptor Tyrosine Kinase	13.61152554
94	STAT3	Signal Transducer And Activator Of Transcription 3	13.60372257
95	PRKAR1A	Protein Kinase CAMP-Dependent Type I Regulatory Subunit Alpha	13.56693172
96	RET	Ret Proto-Oncogene	13.53953171
97	ATP7A	ATPase Copper Transporting Alpha	13.53215408
98	IL10	Interleukin 10	13.47346115
99	PTGS2	Prostaglandin-Endoperoxide Synthase 2	13.25045967
100	IGF2	Insulin Like Growth Factor 2	13.19153404
101	APC	APC Regulator Of WNT Signaling Pathway	13.05152607
102	CDKN2B	Cyclin Dependent Kinase Inhibitor 2B	13.02922535
103	BIRC5	Baculoviral IAP Repeat Containing 5	13.02822208
104	IL2	Interleukin 2	12.97760677
105	KIT	KIT Proto-Oncogene, Receptor Tyrosine Kinase	12.95439243
106	MYOCD	Myocardin	12.8907156
107	MIR141	MicroRNA 141	12.87022686
108	FAS	Fas Cell Surface Death Receptor	12.81436157
109	CD274	CD274 Molecule	12.71169281
110	MIR182	MicroRNA 182	12.67317581
111	CBS	Cystathionine Beta-Synthase	12.61242771
112	MTR	5-Methyltetrahydrofolate-Homocysteine Methyltransferase	12.53116798
113	GNAS	GNAS Complex Locus	12.47575951
114	AURKA	Aurora Kinase A	12.43021774
115	MIR34A	MicroRNA 34a	12.37129974
116	IFNG	Interferon Gamma	12.29374886
117	SPAST	Spastin	12.18548012
118	FLNA	Filamin A	12.11936188
119	DNMT1	DNA Methyltransferase 1	11.97451878
120	CYP1A2	Cytochrome P450 Family 1 Subfamily A Member 2	11.90333748
121	CDK4	Cyclin Dependent Kinase 4	11.83288479
122	TRPV4	Transient Receptor Potential Cation Channel Subfamily V Member 4	11.82336044
123	IL4	Interleukin 4	11.64309502
124	WWOX	WW Domain Containing Oxidoreductase	11.62675953
125	HIF1A	Hypoxia Inducible Factor 1 Subunit Alpha	11.62062931
126	PRPS1	Phosphoribosyl Pyrophosphate Synthetase 1	11.59241009
127	MIR200A	MicroRNA 200a	11.53462505

128	MIR125A	MicroRNA 125a	11.52721405
129	KCNMA1	Potassium Calcium-Activated Channel Subfamily M Alpha 1	11.47454262
130	PPARG	Peroxisome Proliferator Activated Receptor Gamma	11.41087627
131	MIR126	MicroRNA 126	11.39998531
132	CTLA4	Cytotoxic T-Lymphocyte Associated Protein 4	11.38374901
133	IGF1	Insulin Like Growth Factor 1	11.36391258
134	MIR199A1	MicroRNA 199a-1	11.31663227
135	MLH1	MutL Homolog 1	11.31365299
136	MIR30A	MicroRNA 30a	11.27071095
137	KLK3	Kallikrein Related Peptidase 3	11.24741745
138	SOD2	Superoxide Dismutase 2	11.24095631
139	MIR200C	MicroRNA 200c	11.23779964
140	PTCH1	Patched 1	11.15959549
141	STAT1	Signal Transducer And Activator Of Transcription 1	11.13028336
142	MIR142	MicroRNA 142	11.11338711
143	IL1A	Interleukin 1 Alpha	11.10774803
144	TAC1	Tachykinin Precursor 1	11.08838844
145	CYP2D6	Cytochrome P450 Family 2 Subfamily D Member 6	11.00261593
146	EZH2	Enhancer Of Zeste 2 Polycomb Repressive Complex 2 Subunit	10.96627808
147	NFKB1	Nuclear Factor Kappa B Subunit 1	10.92402363
148	MIR214	MicroRNA 214	10.91706371
149	ABCB1	ATP Binding Cassette Subfamily B Member 1	10.86792278
150	FASLG	Fas Ligand	10.86278629
151	BDNF-AS	BDNF Antisense RNA	10.8389616
152	EGR1	Early Growth Response 1	10.80992699
153	LEP	Leptin	10.73168945
154	MIR224	MicroRNA 224	10.72402477
155	SPG7	SPG7 Matrix AAA Peptidase Subunit, Paraplegin	10.71899986
156	TGFBR1	Transforming Growth Factor Beta Receptor 1	10.69599342
157	TLR4	Toll Like Receptor 4	10.68779087
158	GBE1	1,4-Alpha-Glucan Branching Enzyme 1	10.63902283
159	P2RX1	Purinergic Receptor P2X 1	10.55162716
160	MMP14	Matrix Metallopeptidase 14	10.53715229
161	MIR140	MicroRNA 140	10.51496029
162	AMFR	Autocrine Motility Factor Receptor	10.50532246
163	AP5Z1	Adaptor Related Protein Complex 5 Subunit Zeta 1	10.4559412
164	MIR9-1	MicroRNA 9-1	10.43960571
165	E2F1	E2F Transcription Factor 1	10.39669418
166	SQSTM1	Sequestosome 1	10.36565304
167	CHRM1	Cholinergic Receptor Muscarinic 1	10.3633194

168	SPG11	SPG11 Vesicle Trafficking Associated, Spatacsin	10.35773277
169	CDK6	Cyclin Dependent Kinase 6	10.35108185
170	MIR193A	MicroRNA 193a	10.34923172
171	TSC2	TSC Complex Subunit 2	10.32222748
172	FN1	Fibronectin 1	10.29703999
173	RAB27A	RAB27A, Member RAS Oncogene Family	10.27454567
174	CRP	C-Reactive Protein	10.23158264
175	TBP	TATA-Box Binding Protein	10.20846081
176	CSF3	Colony Stimulating Factor 3	10.20552063
177	HPRT1	Hypoxanthine Phosphoribosyltransferase 1	10.20500278
178	INS	Insulin	10.16741371
179	CA9	Carbonic Anhydrase 9	10.07955456
180	IL1B	Interleukin 1 Beta	10.07497406
181	PIK3R1	Phosphoinositide-3-Kinase Regulatory Subunit 1	10.06760979
182	MAPK14	Mitogen-Activated Protein Kinase 14	10.06731415
183	MEN1	Menin 1	10.05972004
184	ZFYVE26	Zinc Finger FYVE-Type Containing 26	10.04606724
185	ALB	Albumin	10.03470707
186	LMNA	Lamin A/C	10.03179359
187	CDKN1C	Cyclin Dependent Kinase Inhibitor 1C	10.01137257
188	PARP1	Poly(ADP-Ribose) Polymerase 1	10.000144
189	IFNA1	Interferon Alpha 1	9.948863983
190	NUMA1	Nuclear Mitotic Apparatus Protein 1	9.946557999
191	CYP2E1	Cytochrome P450 Family 2 Subfamily E Member 1	9.931610107
192	TLR2	Toll Like Receptor 2	9.922103882
193	SDHB	Succinate Dehydrogenase Complex Iron Sulfur Subunit B	9.905913353
194	MIR15A	MicroRNA 15a	9.885965347
195	FGF2	Fibroblast Growth Factor 2	9.863827705
196	MMP7	Matrix Metallopeptidase 7	9.779444695
197	ATL1	Atlastin GTPase 1	9.763801575
198	ALDH18A1	Aldehyde Dehydrogenase 18 Family Member A1	9.717752457
199	MIR139	MicroRNA 139	9.697516441
200	MIR296	MicroRNA 296	9.694735527
201	CYP3A4	Cytochrome P450 Family 3 Subfamily A Member 4	9.642960548
202	MTHFR	Methylenetetrahydrofolate Reductase	9.59314537
203	DBH	Dopamine Beta-Hydroxylase	9.583321571
204	MIR149	MicroRNA 149	9.57437706
205	MIR146A	MicroRNA 146a	9.570455551
206	JUN	Jun Proto-Oncogene, AP-1 Transcription Factor Subunit	9.569101334
207	IGF1R	Insulin Like Growth Factor 1 Receptor	9.566322327
208	CXCL10	C-X-C Motif Chemokine Ligand 10	9.532968521
209	BDNF	Brain Derived Neurotrophic Factor	9.520284653

210	RUNX3	RUNX Family Transcription Factor 3	9.505591393
211	P2RX2	Purinergic Receptor P2X 2	9.462591171
212	ESR1	Estrogen Receptor 1	9.456721306
213	ALK	ALK Receptor Tyrosine Kinase	9.445775032
214	ADAR	Adenosine Deaminase RNA Specific	9.434350967
215	IFNA2	Interferon Alpha 2	9.427321434
216	CDH2	Cadherin 2	9.401155472
217	MIR27A	MicroRNA 27a	9.379321098
218	MPO	Myeloperoxidase	9.37273407
219	CALR	Calreticulin	9.366990089
220	HSPD1	Heat Shock Protein Family D (Hsp60) Member 1	9.355511665
221	CTSD	Cathepsin D	9.316469193
222	NR4A2	Nuclear Receptor Subfamily 4 Group A Member 2	9.25984478
223	ACE	Angiotensin I Converting Enzyme	9.222753525
224	SOX4	SRY-Box Transcription Factor 4	9.222330093
225	IDO1	Indoleamine 2,3-Dioxygenase 1	9.2125597
226	MIR361	MicroRNA 361	9.19629097
227	PIK3CG	Phosphatidylinositol-4,5-Bisphosphate 3-Kinase Catalytic Subunit Gamma	9.186042786
228	MIR93	MicroRNA 93	9.179195404
229	WASHC5	WASH Complex Subunit 5	9.167167664
230	MMP3	Matrix Metallopeptidase 3	9.122851372
231	RYR1	Ryanodine Receptor 1	9.120835304
232	TIMP1	TIMP Metallopeptidase Inhibitor 1	9.082429886
233	CCL5	C-C Motif Chemokine Ligand 5	9.076491356
234	SMAD3	SMAD Family Member 3	9.032464027
235	MIR183	MicroRNA 183	9.030385971
236	CYP7B1	Cytochrome P450 Family 7 Subfamily B Member 1	9.030223846
237	HSPB1	Heat Shock Protein Family B (Small) Member 1	8.996863365
238	COL1A1	Collagen Type I Alpha 1 Chain	8.992907524
239	TWIST1	Twist Family BHLH Transcription Factor 1	8.980301857
240	ACTA2	Actin Alpha 2, Smooth Muscle	8.957530975
241	TGFBR2	Transforming Growth Factor Beta Receptor 2	8.95236969
242	CLU	Clusterin	8.928765297
243	SPARC	Secreted Protein Acidic And Cysteine Rich	8.921042442
244	SUFU	SUFU Negative Regulator Of Hedgehog Signaling	8.920761108
245	RAF1	Raf-1 Proto-Oncogene, Serine/Threonine Kinase	8.907333374
246	ELN	Elastin	8.902405739
247	KDR	Kinase Insert Domain Receptor	8.890216827
248	SYTL1	Synaptotagmin Like 1	8.87156868
249	MMP12	Matrix Metallopeptidase 12	8.869950294
250	EMSLR	E2F1 MRNA Stabilizing LncRNA	8.849985123

251	RHOA	Ras Homolog Family Member A	8.824474335
252	C12orf57	Chromosome 12 Open Reading Frame 57	8.822432518
253	HMOX1	Heme Oxygenase 1	8.808249474
254	XIAP	X-Linked Inhibitor Of Apoptosis	8.781267166
255	MIR155	MicroRNA 155	8.772217751
256	PRKCA	Protein Kinase C Alpha	8.723865509
257	MIR22	MicroRNA 22	8.699354172
258	TSHR	Thyroid Stimulating Hormone Receptor	8.697491646
259	STK11	Serine/Threonine Kinase 11	8.692640305
260	KCNQ1OT1	KCNQ1 Opposite Strand/Antisense Transcript 1	8.688685417
261	VHL	Von Hippel-Lindau Tumor Suppressor	8.682731628
262	ABCC1	ATP Binding Cassette Subfamily C Member 1 (ABCC1 Blood Group)	8.642754555
263	ABCC2	ATP Binding Cassette Subfamily C Member 2	8.636000633
264	GUCY2C	Guanylate Cyclase 2C	8.604590416
265	KIF5A	Kinesin Family Member 5A	8.587215424
266	MIR101-1	MicroRNA 101-1	8.564650536
267	NRG1	Neuregulin 1	8.548268318
268	MIR490	MicroRNA 490	8.547908783
269	NIPA1	NIPA Magnesium Transporter 1	8.541380882
270	HSPA4	Heat Shock Protein Family A (Hsp70) Member 4	8.509992599
271	SHH	Sonic Hedgehog Signaling Molecule	8.503799438
272	SDHA	Succinate Dehydrogenase Complex Flavoprotein Subunit A	8.48524189
273	SYP	Synaptophysin	8.479494095
274	RTN2	Reticulon 2	8.475845337
275	ITGB1	Integrin Subunit Beta 1	8.470767975
276	CXADR	CXADR Ig-Like Cell Adhesion Molecule	8.469581604
277	CD40	CD40 Molecule	8.46096611
278	NOS2	Nitric Oxide Synthase 2	8.455617905
279	TIMP3	TIMP Metallopeptidase Inhibitor 3	8.449085236
280	CSF2	Colony Stimulating Factor 2	8.438710213
281	SERPINE1	Serpin Family E Member 1	8.431157112
282	LTA	Lymphotoxin Alpha	8.428641319
283	MIR106B	MicroRNA 106b	8.404234886
284	IL2RA	Interleukin 2 Receptor Subunit Alpha	8.390965462
285	MIR144	MicroRNA 144	8.388886452
286	MECP2	Methyl-CpG Binding Protein 2	8.352576256
287	SLC2A1	Solute Carrier Family 2 Member 1	8.321582794
288	IL18	Interleukin 18	8.297541618
289	MT-ATP8	Mitochondrially Encoded ATP Synthase Membrane Subunit 8	8.261869431
290	MIR335	MicroRNA 335	8.237888336
291	MIR210	MicroRNA 210	8.195246696
292	COL1A2	Collagen Type I Alpha 2 Chain	8.195171356
293	DPYD	Dihydropyrimidine Dehydrogenase	8.183757782

294	ENO2	Enolase 2	8.182011604
295	ABCG2	ATP Binding Cassette Subfamily G Member 2 (JR Blood Group)	8.172688484
296	NEAT1	Nuclear Paraspeckle Assembly Transcript 1	8.167209625
297	MIR29A	MicroRNA 29a	8.163052559
298	MIR330	MicroRNA 330	8.151996613
299	HLA-A	Major Histocompatibility Complex, Class I, A	8.139610291
300	SNHG1	Small Nucleolar RNA Host Gene 1	8.137577057
301	PLK1	Polo Like Kinase 1	8.128870964
302	TACR1	Tachykinin Receptor 1	8.11991024
303	ICAM1	Intercellular Adhesion Molecule 1	8.087004662
304	MIR29B1	MicroRNA 29b-1	8.04042244
305	SMTN	Smoothelin	8.040124893
306	SOD1	Superoxide Dismutase 1	8.034563065
307	PDE4A	Phosphodiesterase 4A	7.987630844
308	DHFR	Dihydrofolate Reductase	7.977419853
309	MIR125B1	MicroRNA 125b-1	7.974714279
310	RAB27B	RAB27B, Member RAS Oncogene Family	7.971116066
311	MAP2K2	Mitogen-Activated Protein Kinase Kinase 2	7.963947296
312	SYTL2	Synaptotagmin Like 2	7.958572865
313	IGF2R	Insulin Like Growth Factor 2 Receptor	7.950766563
314	CFH	Complement Factor H	7.928217411
315	MIR146B	MicroRNA 146b	7.91657114
316	ADARB1	Adenosine Deaminase RNA Specific B1	7.913731098
317	CAPN1	Calpain 1	7.880010605
318	NGFR	Nerve Growth Factor Receptor	7.869106293
319	CDK2	Cyclin Dependent Kinase 2	7.861065388
320	MIR338	MicroRNA 338	7.838693619
321	CCL21	C-C Motif Chemokine Ligand 21	7.831861973
322	KNG1	Kininogen 1	7.823246956
323	GLI1	GLI Family Zinc Finger 1	7.818822384
324	COL3A1	Collagen Type III Alpha 1 Chain	7.801958084
325	RREB1	Ras Responsive Element Binding Protein 1	7.798368454
326	MIR204	MicroRNA 204	7.772398472
327	FOS	Fos Proto-Oncogene, AP-1 Transcription Factor Subunit	7.757498741
328	MIR129-1	MicroRNA 129-1	7.737780571
329	PLP1	Proteolipid Protein 1	7.721321106
330	MIR124-1	MicroRNA 124-1	7.69022131
331	CDH13	Cadherin 13	7.683625221
332	MIR1247	MicroRNA 1247	7.683561325
333	PPIG	Peptidylprolyl Isomerase G	7.68266201
334	HSPA8	Heat Shock Protein Family A (Hsp70) Member 8	7.671189785
335	MIR186	MicroRNA 186	7.639927387
336	IRF1	Interferon Regulatory Factor 1	7.636219025

337	MIR582	MicroRNA 582	7.612500191
338	SYNPO2	Synaptopodin 2	7.605557442
339	REEP1	Receptor Accessory Protein 1	7.570307255
340	CRH	Corticotropin Releasing Hormone	7.566952705
341	SST	Somatostatin	7.563707352
342	MIR590	MicroRNA 590	7.520152092
343	MIR193B	MicroRNA 193b	7.518671513
344	RAG1	Recombination Activating 1	7.490258217
345	STAT5B	Signal Transducer And Activator Of Transcription 5B	7.477138996
346	TNFRSF1A	TNF Receptor Superfamily Member 1A	7.473036289
347	SNCA	Synuclein Alpha	7.473032475
348	KCNJ5	Potassium Inwardly Rectifying Channel Subfamily J Member 5	7.448897362
349	HLA-DRB1	Major Histocompatibility Complex, Class II, DR Beta 1	7.448769569
350	MIR675	MicroRNA 675	7.406268597
351	FUS	FUS RNA Binding Protein	7.392852306
352	COMT	Catechol-O-Methyltransferase	7.38686657
353	MIR370	MicroRNA 370	7.375450611
354	MIR16-1	MicroRNA 16-1	7.324776649
355	MIR148A	MicroRNA 148a	7.315613747
356	NOTCH3	Notch Receptor 3	7.30753231
357	ATXN2	Ataxin 2	7.283229351
358	RELA	RELA Proto-Oncogene, NF-KB Subunit	7.274889469
359	PDE5A	Phosphodiesterase 5A	7.271930218
360	SOCS1	Suppressor Of Cytokine Signaling 1	7.235198021
361	FOXP3	Forkhead Box P3	7.220809937
362	MIRLET7C	MicroRNA Let-7c	7.210630894
363	ADA	Adenosine Deaminase	7.20331192
364	FA2H	Fatty Acid 2-Hydroxylase	7.201094627
365	MIR106A	MicroRNA 106a	7.186614513
366	CHKA	Choline Kinase Alpha	7.168582916
367	VIP	Vasoactive Intestinal Peptide	7.163043022
368	VWF	Von Willebrand Factor	7.158007622
369	MIR423	MicroRNA 423	7.156267643
370	MIR150	MicroRNA 150	7.150052071
371	PIK3CB	Phosphatidylinositol-4,5-Bisphosphate 3-Kinase Catalytic Subunit Beta	7.144090652
372	CDR1-AS	CDR1 Antisense RNA	7.113556385
373	CERNA3	Competing Endogenous LncRNA 3 For MiR-645	7.106686592
374	SFRP2	Secreted Frizzled Related Protein 2	7.064043045
375	MIR424	MicroRNA 424	7.028445244
376	PAX8	Paired Box 8	7.013747692
377	EDN1	Endothelin 1	6.988413811
378	CCK	Cholecystokinin	6.954341412
379	COL4A1	Collagen Type IV Alpha 1 Chain	6.928639412
380	MIR211	MicroRNA 211	6.902544022
381	PKD1	Polycystin 1, Transient Receptor Potential Channel Interacting	6.862165451

382	LOX	Lysyl Oxidase	6.854808807
383	CDC73	Cell Division Cycle 73	6.842778683
384	MIR130B	MicroRNA 130b	6.796895981
385	APOA1	Apolipoprotein A1	6.79651022
386	POLG	DNA Polymerase Gamma, Catalytic Subunit	6.791786194
387	MT-ND1	Mitochondrially Encoded NADH:Ubiquinone Oxidoreductase Core Subunit 1	6.764709473
388	CDH11	Cadherin 11	6.761809349
389	NPY	Neuropeptide Y	6.746472359
390	REEP2	Receptor Accessory Protein 2	6.717740059
391	FMR1	Fragile X Messenger Ribonucleoprotein 1	6.712791443
392	KIF1A	Kinesin Family Member 1A	6.702580929
393	CCL26	C-C Motif Chemokine Ligand 26	6.678730011
394	DNAH8	Dynein Axonemal Heavy Chain 8	6.634932995
395	NKX2-1	NK2 Homeobox 1	6.578965187
396	KCNJ11	Potassium Inwardly Rectifying Channel Subfamily J Member 11	6.571888924
397	ATXN10	Ataxin 10	6.561067581
398	UCHL1	Ubiquitin C-Terminal Hydrolase L1	6.559855938
399	ATP13A2	ATPase Cation Transporting 13A2	6.494360924
400	MIR181A1	MicroRNA 181a-1	6.490416527
401	SPART	Spartin	6.483587742
402	ADRA1A	Adrenoceptor Alpha 1A	6.481346607
403	MLPH	Melanophilin	6.44736433
404	PDGFRA	Platelet Derived Growth Factor Receptor Alpha	6.43400383
405	HLA-DQB1	Major Histocompatibility Complex, Class II, DQ Beta 1	6.422745705
406	TACR2	Tachykinin Receptor 2	6.407706261
407	PDGFRB	Platelet Derived Growth Factor Receptor Beta	6.404828072
408	ATXN3	Ataxin 3	6.404154778
409	LINC01672	Long Intergenic Non-Protein Coding RNA 1672	6.403829575
410	CD8A	CD8 Subunit Alpha	6.391065121
411	MAPT	Microtubule Associated Protein Tau	6.354795456
412	TRPM8	Transient Receptor Potential Cation Channel Subfamily M Member 8	6.332765579
413	CD4	CD4 Molecule	6.315898895
414	MT-CYB	Mitochondrially Encoded Cytochrome B	6.289596558
415	MIR432	MicroRNA 432	6.231893063
416	POR	Cytochrome P450 Oxidoreductase	6.211771488
417	MIR485	MicroRNA 485	6.190268517
418	APOE	Apolipoprotein E	6.181176662
419	SDHD	Succinate Dehydrogenase Complex Subunit D	6.179691315
420	REN	Renin	6.177269936
421	TNFRSF11B	TNF Receptor Superfamily Member 11b	6.162265778

422	ITGAE	Integrin Subunit Alpha E	6.159463882
423	TNFRSF11A	TNF Receptor Superfamily Member 11a	6.158586979
424	CHGA	Chromogranin A	6.127677441
425	EPHB2	EPH Receptor B2	6.122922421
426	MIR133A1	MicroRNA 133a-1	6.114463329
427	FRAS1	Fraser Extracellular Matrix Complex Subunit 1	5.993648052
428	DICER1	Dicer 1, Ribonuclease III	5.992969036
429	UBAP1	Ubiquitin Associated Protein 1	5.983516693
430	LMNB1	Lamin B1	5.975577354
431	MIR24-1	MicroRNA 24-1	5.951217651
432	IL13	Interleukin 13	5.939496994
433	MIR135A1	MicroRNA 135a-1	5.921428204
434	SIGMAR1	Sigma Non-Opioid Intracellular Receptor 1	5.882245064
435	ALMS1	ALMS1 Centrosome And Basal Body Associated Protein	5.877169609
436	PIK3CD	Phosphatidylinositol-4,5-Bisphosphate 3-Kinase Catalytic Subunit Delta	5.876617432
437	S100B	S100 Calcium Binding Protein B	5.871621132
438	MPZ	Myelin Protein Zero	5.857411385
439	MIR19A	MicroRNA 19a	5.845404148
440	ABCC8	ATP Binding Cassette Subfamily C Member 8	5.775319099
441	PLA2G6	Phospholipase A2 Group VI	5.765940189
442	BBS5	Bardet-Biedl Syndrome 5	5.757964611
443	PRL	Prolactin	5.749806404
444	SCAPER	S-Phase Cyclin A Associated Protein In The ER	5.742313385
445	BGLAP	Bone Gamma-Carboxyglutamate Protein	5.726475716
446	COQ2	Coenzyme Q2, Polyprenyltransferase	5.711670876
447	TAGLN	Transgelin	5.699251652
448	TH	Tyrosine Hydroxylase	5.695417404
449	ZEB2	Zinc Finger E-Box Binding Homeobox 2	5.69034338
450	CEACAM5	CEA Cell Adhesion Molecule 5	5.669474602
451	ESR2	Estrogen Receptor 2	5.658404827
452	DRD4	Dopamine Receptor D4	5.647043705
453	SPP1	Secreted Phosphoprotein 1	5.629104614
454	VDR	Vitamin D Receptor	5.629089355
455	GRN	Granulin Precursor	5.610721111
456	CALCA	Calcitonin Related Polypeptide Alpha	5.582551956
457	KCNK2	Potassium Two Pore Domain Channel Subfamily K Member 2	5.581084251
458	GATA6	GATA Binding Protein 6	5.535663605
459	ABCD1	ATP Binding Cassette Subfamily D Member 1	5.505434036
460	CACNA1A	Calcium Voltage-Gated Channel Subunit Alpha1 A	5.502730846
461	PDGFB	Platelet Derived Growth Factor Subunit B	5.492691994

462	PRKACA	Protein Kinase CAMP-Activated Catalytic Subunit Alpha	5.473424911
463	MIR26A1	MicroRNA 26a-1	5.467362404
464	IL7R	Interleukin 7 Receptor	5.459215641
465	POMC	Proopiomelanocortin	5.453793526
466	MIR19B1	MicroRNA 19b-1	5.450111866
467	NOTCH1	Notch Receptor 1	5.441853523
468	GBA1	Glucosylceramidase Beta 1	5.424310684
469	LIFR	LIF Receptor Subunit Alpha	5.405078411
470	MIR181B1	MicroRNA 181b-1	5.399286747
471	MIR605	MicroRNA 605	5.397348404
472	TRPA1	Transient Receptor Potential Cation Channel Subfamily A Member 1	5.395830154
473	MIR34C	MicroRNA 34c	5.382145882
474	KMT2A	Lysine Methyltransferase 2A	5.363696575
475	NF2	NF2, Moesin-Ezrin-Radixin Like (MERLIN) Tumor Suppressor	5.354445457
476	NOS3	Nitric Oxide Synthase 3	5.35051918
477	LRRK2	Leucine Rich Repeat Kinase 2	5.331939697
478	MIR18A	MicroRNA 18a	5.326282501
479	APRT	Adenine Phosphoribosyltransferase	5.320020199
480	BRIP1	BRCA1 Interacting Helicase 1	5.311450481
481	MIR320A	MicroRNA 320a	5.308421135
482	TFRC	Transferrin Receptor	5.294699669
483	TMX2-CTNND1	TMX2-CTNND1 Readthrough (NMD Candidate)	5.281003952
484	ATXN8OS	ATXN8 Opposite Strand LncRNA	5.279417992
485	CLCNKB	Chloride Voltage-Gated Channel Kb	5.272472858
486	BAP1	BRCA1 Associated Protein 1	5.242296219
487	GFAP	Glial Fibrillary Acidic Protein	5.228910923
488	MIR532	MicroRNA 532	5.226261139
489	P3H4	Prolyl 3-Hydroxylase Family Member 4 (Inactive)	5.220890999
490	VCP	Valosin Containing Protein	5.193260193
491	ERLIN2	ER Lipid Raft Associated 2	5.186173439
492	GC	GC Vitamin D Binding Protein	5.177186489
493	GNRH1	Gonadotropin Releasing Hormone 1	5.147174835
494	TGFB2	Transforming Growth Factor Beta 2	5.112332344
495	AHR	Aryl Hydrocarbon Receptor	5.103102207
496	CKAP4	Cytoskeleton Associated Protein 4	5.099663258
497	MIR192	MicroRNA 192	5.093193054
498	KCNC3	Potassium Voltage-Gated Channel Subfamily C Member 3	5.09087944
499	IL17A	Interleukin 17A	5.076945305
500	ACHE	Acetylcholinesterase (Yt Blood Group)	5.06935215
501	MIRLET7B	MicroRNA Let-7b	4.994641304
502	ADRB2	Adrenoceptor Beta 2	4.990652084
503	FBN1	Fibrillin 1	4.982606411
504	MIR328	MicroRNA 328	4.978531361
505	MIR7-1	MicroRNA 7-1	4.977281094

506	FARS2	Phenylalanyl-TRNA Synthetase 2, Mitochondrial	4.976791382
507	HTR2A	5-Hydroxytryptamine Receptor 2A	4.936878204
508	IL5	Interleukin 5	4.931728363
509	DUSP1	Dual Specificity Phosphatase 1	4.928877831
510	SLC12A3	Solute Carrier Family 12 Member 3	4.927361488
511	GAL	Galanin And GMAP Prepropeptide	4.916282654
512	NPPA	Natriuretic Peptide A	4.911858559
513	SMAD2	SMAD Family Member 2	4.899512768
514	LINC-ROR	Long Intergenic Non-Protein Coding RNA, Regulator Of Reprogramming	4.885946274
515	SOD2-OT1	SOD2 Overlapping Transcript 1	4.87524128
516	PLAGL1	PLAG1 Like Zinc Finger 1	4.866183281
517	MIR196A1	MicroRNA 196a-1	4.844545364
518	CRHR1	Corticotropin Releasing Hormone Receptor 1	4.83769989
519	SELE	Selectin E	4.820450783
520	HLA-B	Major Histocompatibility Complex, Class I, B	4.80022049
521	CYP2C9	Cytochrome P450 Family 2 Subfamily C Member 9	4.792359829
522	MIR324	MicroRNA 324	4.792358398
523	APOB	Apolipoprotein B	4.784856796
524	CYP19A1	Cytochrome P450 Family 19 Subfamily A Member 1	4.758364677
525	ATP1A3	ATPase Na+/K+ Transporting Subunit Alpha 3	4.756531239
526	NCAM1	Neural Cell Adhesion Molecule 1	4.740571022
527	CPT1C	Carnitine Palmitoyltransferase 1C	4.735313416
528	PRNP	Prion Protein (Kanno Blood Group)	4.718419075
529	B4GALNT1	Beta-1,4-N-Acetyl-Galactosaminyltransferase 1	4.694515228
530	SHC1	SHC Adaptor Protein 1	4.690178871
531	NPPB	Natriuretic Peptide B	4.689472675
532	DRD2	Dopamine Receptor D2	4.68892765
533	MIR877	MicroRNA 877	4.685256004
534	KIF1B	Kinesin Family Member 1B	4.680944443
535	NR3C1	Nuclear Receptor Subfamily 3 Group C Member 1	4.679380894
536	MTRR	5-Methyltetrahydrofolate-Homocysteine Methyltransferase Reductase	4.67779541
537	ALS2	Alsin Rho Guanine Nucleotide Exchange Factor ALS2	4.677406788
538	GSK3B	Glycogen Synthase Kinase 3 Beta	4.673549652
539	GPT	Glutamic--Pyruvic Transaminase	4.664628506
540	FOXO3	Forkhead Box O3	4.660191536
541	SLC11A1	Solute Carrier Family 11 Member 1	4.659795284
542	NR5A1	Nuclear Receptor Subfamily 5 Group A Member 1	4.659638405
543	PNPLA6	Patatin Like Phospholipase Domain Containing 6	4.65570116

544	APTX	Aprataxin	4.653362274
545	NFKBIA	NFKB Inhibitor Alpha	4.648691177
546	SETX	Senataxin	4.627292633
547	SOX2	SRY-Box Transcription Factor 2	4.610429287
548	KITLG	KIT Ligand	4.609102249
549	MAPK3	Mitogen-Activated Protein Kinase 3	4.598135948
550	SACS	Sacsin Molecular Chaperone	4.597770214
551	MIR486-1	MicroRNA 486-1	4.589288235
552	ADH1C	Alcohol Dehydrogenase 1C (Class I), Gamma Polypeptide	4.583482265
553	SLC6A3	Solute Carrier Family 6 Member 3	4.582616806
554	MIR455	MicroRNA 455	4.577167034
555	TCHP	Trichoplein Keratin Filament Binding	4.568730831
556	FAT4	FAT Atypical Cadherin 4	4.56581974
557	EIF2AK3	Eukaryotic Translation Initiation Factor 2 Alpha Kinase 3	4.561639309
558	IL1RN	Interleukin 1 Receptor Antagonist	4.553857803
559	GRIN2B	Glutamate Ionotropic Receptor NMDA Type Subunit 2B	4.54585886
560	CD80	CD80 Molecule	4.541695595
561	KPNA3	Karyopherin Subunit Alpha 3	4.522476196
562	EYA1	EYA Transcriptional Coactivator And Phosphatase 1	4.497051716
563	SCN1A	Sodium Voltage-Gated Channel Alpha Subunit 1	4.470125675
564	B2M	Beta-2-Microglobulin	4.469901562
565	HDAC9	Histone Deacetylase 9	4.448187351
566	KLC2	Kinesin Light Chain 2	4.447086811
567	SRGAP1	SLIT-ROBO Rho GTPase Activating Protein 1	4.444851875
568	MIR34B	MicroRNA 34b	4.430164814
569	PTPRC	Protein Tyrosine Phosphatase Receptor Type C	4.412378311
570	PNOC	Prepronociceptin	4.402094841
571	TMEM63C	Transmembrane Protein 63C	4.398738384
572	DDHD2	DDHD Domain Containing 2	4.398302555
573	PKHD1	PKHD1 Ciliary IPT Domain Containing Fibrocystin/Polyductin	4.398132324
574	MAP1B	Microtubule Associated Protein 1B	4.394942284
575	CHRM4	Cholinergic Receptor Muscarinic 4	4.386351585
576	CREB1	CAMP Responsive Element Binding Protein 1	4.381238937
577	MIR92A1	MicroRNA 92a-1	4.37749052
578	OPTN	Optineurin	4.360748291
579	BMP6	Bone Morphogenetic Protein 6	4.359873295
580	HDAC1	Histone Deacetylase 1	4.353591442
581	VCAM1	Vascular Cell Adhesion Molecule 1	4.349134445
582	SYNE1	Spectrin Repeat Containing Nuclear Envelope Protein 1	4.347999573
583	MKS1	MKS Transition Zone Complex Subunit 1	4.329752445

584	MOG	Myelin Oligodendrocyte Glycoprotein	4.328773975
585	IFT172	Intraflagellar Transport 172	4.314022064
586	LINC02605	Long Intergenic Non-Protein Coding RNA 2605	4.31151247
587	F2	Coagulation Factor II, Thrombin	4.307951927
588	PTH	Parathyroid Hormone	4.303068638
589	GJC1	Gap Junction Protein Gamma 1	4.301795959
590	ARHGEF2	Rho/Rac Guanine Nucleotide Exchange Factor 2	4.29050827
591	ASXL1	ASXL Transcriptional Regulator 1	4.289357185
592	PIGQ	Phosphatidylinositol Glycan Anchor Biosynthesis Class Q	4.286779404
593	PINK1	PTEN Induced Kinase 1	4.273487091
594	MT-TT	Mitochondrially Encoded TRNA-Thr (ACN)	4.265141487
595	BSCL2	BSCL2 Lipid Droplet Biogenesis Associated, Seipin	4.253988266
596	ZEB1	Zinc Finger E-Box Binding Homeobox 1	4.197946548
597	KIF5B	Kinesin Family Member 5B	4.188745499
598	KIF1C	Kinesin Family Member 1C	4.169892311
599	KCNJ18	Potassium Inwardly Rectifying Channel Subfamily J Member 18	4.168945789
600	MIF	Macrophage Migration Inhibitory Factor	4.168557167
601	PSIP1	PC4 And SRSF1 Interacting Protein 1	4.157614708
602	MIRLET7A1	MicroRNA Let-7a-1	4.156060219
603	ARNT	Aryl Hydrocarbon Receptor Nuclear Translocator	4.154062271
604	VPS11	VPS11 Core Subunit Of CORVET And HOPS Complexes	4.153651714
605	DCN	Decorin	4.148693085
606	RAC1	Rac Family Small GTPase 1	4.135187149
607	SMAD4	SMAD Family Member 4	4.133276939
608	PRKN	Parkin RBR E3 Ubiquitin Protein Ligase	4.125021458
609	PLAT	Plasminogen Activator, Tissue Type	4.117643833
610	MIR187	MicroRNA 187	4.116476059
611	MYO5A	Myosin VA	4.11227417
612	TRPM7	Transient Receptor Potential Cation Channel Subfamily M Member 7	4.109392166
613	CACNA1C	Calcium Voltage-Gated Channel Subunit Alpha1 C	4.100334167
614	INSR	Insulin Receptor	4.089426994
615	ERLIN1	ER Lipid Raft Associated 1	4.086386681
616	CD36	CD36 Molecule (CD36 Blood Group)	4.084973335
617	GDNF	Glial Cell Derived Neurotrophic Factor	4.08087492
618	SDHC	Succinate Dehydrogenase Complex Subunit C	4.079414368
619	FAR1	Fatty Acyl-CoA Reductase 1	4.071876526
620	PPM1D	Protein Phosphatase, Mg2+/Mn2+ Dependent 1D	4.070816517
621	ZNF687	Zinc Finger Protein 687	4.069939137

622	FZD8	Frizzled Class Receptor 8	4.064897537
623	FOXM1	Forkhead Box M1	4.06108427
624	HLA-G	Major Histocompatibility Complex, Class I, G	4.057835102
625	FLT1	Fms Related Receptor Tyrosine Kinase 1	4.046316147
626	SCARB1	Scavenger Receptor Class B Member 1	4.022326469
627	PI4KA	Phosphatidylinositol 4-Kinase Alpha	4.009814262
628	CDC6	Cell Division Cycle 6	4.006781101
629	HCRT	Hypocretin Neuropeptide Precursor	4.00469923
630	MAG	Myelin Associated Glycoprotein	4.00430727
631	F3	Coagulation Factor III, Tissue Factor	4.002080441
632	AGTR1	Angiotensin II Receptor Type 1	3.998441219
633	HPDL	4-Hydroxyphenylpyruvate Dioxygen-ase Like	3.997234106
634	AFP	Alpha Fetoprotein	3.996422768
635	GJA5	Gap Junction Protein Alpha 5	3.984979391
636	CD86	CD86 Molecule	3.977036238
637	JAK2	Janus Kinase 2	3.975876808
638	PIEZO1	Piezo Type Mechanosensitive Ion Channel Component 1 (Er Blood Group)	3.969176292
639	LDLR	Low Density Lipoprotein Receptor	3.963243723
640	PTPN11	Protein Tyrosine Phosphatase Non-Re-ceptor Type 11	3.957241297
641	RFX6	Regulatory Factor X6	3.953538895
642	HYMAI	Hydatidiform Mole Associated And Imprinted	3.948563576
643	DNASE1	Deoxyribonuclease 1	3.943813324
644	IL4R	Interleukin 4 Receptor	3.937858582
645	WNT7A	Wnt Family Member 7A	3.934176922
646	ARVCF	ARVCF Delta Catenin Family Member	3.933970928
647	PAX6	Paired Box 6	3.930086613
648	SNAP25	Synaptosome Associated Protein 25	3.921075344
649	AXIN1	Axin 1	3.920632362
650	SOCS3	Suppressor Of Cytokine Signaling 3	3.920327663
651	MT-CO3	Mitochondrially Encoded Cytochrome C Oxidase III	3.91618371
652	CDKN2C	Cyclin Dependent Kinase Inhibitor 2C	3.906183481
653	JUND	JunD Proto-Oncogene, AP-1 Transcrip-tion Factor Subunit	3.90518713
654	MSMB	Microseminoprotein Beta	3.903000832
655	PKM	Pyruvate Kinase M1/2	3.89991498
656	UBE4A	Ubiquitination Factor E4A	3.887424707
657	TJP1	Tight Junction Protein 1	3.879132748
658	XRCC5	X-Ray Repair Cross Complementing 5	3.873666048
659	CBL	Cbl Proto-Oncogene	3.871227264
660	NBEA	Neurobeachin	3.869724751
661	HEXB	Hexosaminidase Subunit Beta	3.86870575
662	STX1A	Syntaxin 1A	3.866772175
663	CHRM5	Cholinergic Receptor Muscarinic 5	3.861234426

664	USP8	Ubiquitin Specific Peptidase 8	3.860921383
665	CDH23	Cadherin Related 23	3.858997822
666	COL5A2	Collagen Type V Alpha 2 Chain	3.855956793
667	FZD5	Frizzled Class Receptor 5	3.853668213
668	TPX2	TPX2 Microtubule Nucleation Factor	3.85170269
669	MIR132	MicroRNA 132	3.850936651
670	MIRLET7D	MicroRNA Let-7d	3.845303535
671	OTX1	Orthodenticle Homeobox 1	3.835444927
672	CASR	Calcium Sensing Receptor	3.833278656
673	SSTR2	Somatostatin Receptor 2	3.818601608
674	ATRX	ATRX Chromatin Remodeler	3.809387207
675	XRCC6	X-Ray Repair Cross Complementing 6	3.799344778
676	CXCR3	C-X-C Motif Chemokine Receptor 3	3.799019814
677	CNR1	Cannabinoid Receptor 1	3.797595263
678	CYP17A1	Cytochrome P450 Family 17 Subfamily A Member 1	3.79754281
679	TG	Thyroglobulin	3.783040047
680	DKK1	Dickkopf WNT Signaling Pathway Inhibitor 1	3.779123545
681	ADIPOQ	Adiponectin, C1Q And Collagen Domain Containing	3.778030396
682	CFTR	CF Transmembrane Conductance Regulator	3.767373085
683	USP48	Ubiquitin Specific Peptidase 48	3.760402203
684	NOD2	Nucleotide Binding Oligomerization Domain Containing 2	3.759192467
685	CD40LG	CD40 Ligand	3.754009485
686	TARDBP	TAR DNA Binding Protein	3.744856834
687	DEPDC5	DEP Domain Containing 5, GATOR1 Subcomplex Subunit	3.74041152
688	KCNT1	Potassium Sodium-Activated Channel Subfamily T Member 1	3.74041152
689	TTC21B	Tetratricopeptide Repeat Domain 21B	3.736885786
690	CDK20	Cyclin Dependent Kinase 20	3.73435545
691	CASP2	Caspase 2	3.727600098
692	OXT	Oxytocin/Neurophysin I Prepropeptide	3.721081257
693	THORLNC	Testis Associated Oncogenic LncRNA	3.716084003
694	DPF2	Double PHD Fingers 2	3.711193562
695	SIRT1	Sirtuin 1	3.704559565
696	CCR2	C-C Motif Chemokine Receptor 2	3.692998409
697	PAX3	Paired Box 3	3.688569784
698	CDK5	Cyclin Dependent Kinase 5	3.684593916
699	FCGR2A	Fc Gamma Receptor IIa	3.663537025
700	MIR451A	MicroRNA 451a	3.653112888
701	JAK3	Janus Kinase 3	3.649722338
702	ENTPD1	Ectonucleoside Triphosphate Diphosphohydrolase 1	3.64724803
703	ARL6IP1	ADP Ribosylation Factor Like GTPase 6 Interacting Protein 1	3.639059544
704	TBR1	T-Box Brain Transcription Factor 1	3.637276649

705	SPTLC1	Serine Palmitoyltransferase Long Chain Base Subunit 1	3.63236475
706	MSTO1	Misato Mitochondrial Distribution And Morphology Regulator 1	3.617248535
707	ENPP1	Ectonucleotide Pyrophosphatase/Phosphodiesterase 1	3.601322651
708	BLTP1	Bridge-Like Lipid Transfer Protein Family Member 1	3.571358204
709	KCNQ1	Potassium Voltage-Gated Channel Subfamily Q Member 1	3.568963528
710	AGT	Angiotensinogen	3.568234921
711	NLRP3	NLR Family Pyrin Domain Containing 3	3.567049026
712	ROCK2	Rho Associated Coiled-Coil Containing Protein Kinase 2	3.561450958
713	NTRK1	Neurotrophic Receptor Tyrosine Kinase 1	3.560705423
714	CYP24A1	Cytochrome P450 Family 24 Subfamily A Member 1	3.55370903
715	HTR1A	5-Hydroxytryptamine Receptor 1A	3.546543121
716	TSPO	Translocator Protein	3.545991898
717	CNNM2	Cyclin And CBS Domain Divalent Metal Cation Transport Mediator 2	3.545403481
718	KCNA3	Potassium Voltage-Gated Channel Subfamily A Member 3	3.532369137
719	EPO	Erythropoietin	3.527459383
720	SCARNA5	Small Cajal Body-Specific RNA 5	3.514773607
721	CYP2C19	Cytochrome P450 Family 2 Subfamily C Member 19	3.513844013
722	JAK1	Janus Kinase 1	3.513390541
723	PGR-AS1	PGR Antisense RNA 1	3.509623528
724	SRD5A1	Steroid 5 Alpha-Reductase 1	3.492053986
725	NTRK2	Neurotrophic Receptor Tyrosine Kinase 2	3.483715057
726	LIF	LIF Interleukin 6 Family Cytokine	3.474822998
727	NOS1	Nitric Oxide Synthase 1	3.47330451
728	ENO1	Enolase 1	3.466532707
729	GSR	Glutathione-Disulfide Reductase	3.462486267
730	XDH	Xanthine Dehydrogenase	3.453375101
731	VPS13B	Vacuolar Protein Sorting 13 Homolog B	3.452405691
732	IL6ST	Interleukin 6 Cytokine Family Signal Transducer	3.451899529
733	IL15	Interleukin 15	3.442959785
734	KY	Kyphoscoliosis Peptidase	3.436988115
735	IBA57	Iron-Sulfur Cluster Assembly Factor IBA57	3.42595005
736	GNB5	G Protein Subunit Beta 5	3.423035145
737	ITGB3	Integrin Subunit Beta 3	3.418016195
738	MIR518A1	MicroRNA 518a-1	3.412722111
739	ALPP	Alkaline Phosphatase, Placental	3.412340879
740	ARFGEF1-DT	ARFGEF1 Divergent Transcript	3.407088041

741	ABCB11	ATP Binding Cassette Subfamily B Member 11	3.377521515
742	HTR3A	5-Hydroxytryptamine Receptor 3A	3.374118805
743	AVP	Arginine Vasopressin	3.373222589
744	MIR483	MicroRNA 483	3.367007971
745	NEFL	Neurofilament Light Chain	3.366858006
746	POLR3A	RNA Polymerase III Subunit A	3.364239931
747	GNAS-AS1	GNAS Antisense RNA 1	3.346311808
748	LOC129935068	ATAC-STARR-Seq Lymphoblastoid Active Region 16738	3.346179962
749	SCNN1A	Sodium Channel Epithelial 1 Subunit Alpha	3.338541746
750	SLC16A2	Solute Carrier Family 16 Member 2	3.336807251
751	SLC33A1	Solute Carrier Family 33 Member 1	3.32725215
752	KHDRBS1	KH RNA Binding Domain Containing, Signal Transduction Associated 1	3.326378345
753	SMAD5-AS1	SMAD5 Antisense RNA 1	3.322292805
754	CCR5	C-C Motif Chemokine Receptor 5	3.30676651
755	CNC2	Carney Complex Type 2, Multiple Neoplasia And Lentiginosis	3.304681063
756	SHBG	Sex Hormone Binding Globulin	3.303298712
757	MARS2	Methionyl-TRNA Synthetase 2, Mitochondrial	3.294404507
758	PWAR1	Prader Willi/Angelman Region RNA 1	3.293201685
759	ADRA1D	Adrenoceptor Alpha 1D	3.292731762
760	CNOT1	CCR4-NOT Transcription Complex Subunit 1	3.281057596
761	ABCG8	ATP Binding Cassette Subfamily G Member 8	3.280394554
762	CSF1R	Colony Stimulating Factor 1 Receptor	3.276768208
763	MAOA	Monoamine Oxidase A	3.276229382
764	OPRM1	Opioid Receptor Mu 1	3.271718025
765	MAGED2	MAGE Family Member D2	3.269350767
766	TOP1	DNA Topoisomerase I	3.269083977
767	HNF1A	HNF1 Homeobox A	3.265807152
768	CYP2U1	Cytochrome P450 Family 2 Subfamily U Member 1	3.259660721
769	MVP	Major Vault Protein	3.254324436
770	COX5A	Cytochrome C Oxidase Subunit 5A	3.241804838
771	CHRNA4	Cholinergic Receptor Nicotinic Alpha 4 Subunit	3.2382164
772	PFN1	Profilin 1	3.235701084
773	CGA	Glycoprotein Hormones, Alpha Polypeptide	3.234265804
774	CEP128	Centrosomal Protein 128	3.228888273
775	NIPBL	NIPBL Cohesin Loading Factor	3.22805953
776	HLA-DQA1	Major Histocompatibility Complex, Class II, DQ Alpha 1	3.225153208
777	PSEN1	Presenilin 1	3.224551916
778	ARX	Aristaless Related Homeobox	3.21356678

779	CACNA1G	Calcium Voltage-Gated Channel Subunit Alpha1 G	3.205637932
780	CSNK1D	Casein Kinase 1 Delta	3.197234631
781	TWNK	Twinkle MtDNA Helicase	3.194075108
782	EDNRA	Endothelin Receptor Type A	3.190366268
783	CD79A	CD79a Molecule	3.185859919
784	FAT1	FAT Atypical Cadherin 1	3.16958952
785	HP	Haptoglobin	3.163443089
786	RBL2	RB Transcriptional Corepressor Like 2	3.143792629
787	ARRB2	Arrestin Beta 2	3.142089367
788	ACE2	Angiotensin Converting Enzyme 2	3.136518955
789	FXYD2	FXYD Domain Containing Ion Transport Regulator 2	3.126254082
790	SCD	Stearoyl-CoA Desaturase	3.123457909
791	HFE	Homeostatic Iron Regulator	3.120773792
792	TRC-GCA24-1	TRNA-Cys (GCA) 24-1	3.118340969
793	TRIM28	Tripartite Motif Containing 28	3.114455223
794	PRKAR2B	Protein Kinase CAMP-Dependent Type II Regulatory Subunit Beta	3.112614393
795	RFX1	Regulatory Factor X1	3.1123209
796	PLK2	Polo Like Kinase 2	3.108708382
797	SMAD7	SMAD Family Member 7	3.107439756
798	EIF2AK1	Eukaryotic Translation Initiation Factor 2 Alpha Kinase 1	3.107058048
799	SCN9A	Sodium Voltage-Gated Channel Alpha Subunit 9	3.104973793
800	ELAVL1	ELAV Like RNA Binding Protein 1	3.093752861
801	MLN	Motilin	3.075251102
802	MIR151A	MicroRNA 151a	3.067602634
803	FUT2	Fucosyltransferase 2 (H Blood Group)	3.063421726
804	CNR2	Cannabinoid Receptor 2	3.061490297
805	IL12B	Interleukin 12B	3.059175491
806	SERPINA7	Serpin Family A Member 7	3.056758642
807	TNFRSF1B	TNF Receptor Superfamily Member 1B	3.048461437
808	KCNJ1	Potassium Inwardly Rectifying Channel Subfamily J Member 1	3.048258305
809	SV2A	Synaptic Vesicle Glycoprotein 2A	3.04214716
810	GRIN2A	Glutamate Ionotropic Receptor NMDA Type Subunit 2A	3.0289855
811	CHAT	Choline O-Acetyltransferase	3.023528576
812	GAST	Gastrin	3.019736767
813	LPP	LIM Domain Containing Preferred Translocation Partner In Lipoma	3.017049789
814	CACNA1S	Calcium Voltage-Gated Channel Subunit Alpha1 S	3.015832186
815	SLC6A4	Solute Carrier Family 6 Member 4	3.009745836
816	GAD2	Glutamate Decarboxylase 2	3.002310991
817	TBX1	T-Box Transcription Factor 1	3.000536919
818	TECPR2	Tectonin Beta-Propeller Repeat Containing 2	2.9928689

819	PTPRJ	Protein Tyrosine Phosphatase Receptor Type J	2.991106272
820	TRIM25	Tripartite Motif Containing 25	2.990450144
821	MT-ND2	Mitochondrially Encoded NADH:Ubiquinone Oxidoreductase Core Subunit 2	2.988481045
822	DRD1	Dopamine Receptor D1	2.988200188
823	RAB9B	RAB9B, Member RAS Oncogene Family	2.975209713
824	MBL2	Mannose Binding Lectin 2	2.972638607
825	CXCL5	C-X-C Motif Chemokine Ligand 5	2.970045567
826	GJB1	Gap Junction Protein Beta 1	2.963653088
827	CKB	Creatine Kinase B	2.955230713
828	NTF3	Neurotrophin 3	2.945183277
829	TIA1	TIA1 Cytotoxic Granule Associated RNA Binding Protein	2.943677425
830	MYCN	MYCN Proto-Oncogene, BHLH Transcription Factor	2.941558599
831	CYP11B2	Cytochrome P450 Family 11 Subfamily B Member 2	2.941433191
832	CYLD	CYLD Lysine 63 Deubiquitinase	2.936292887
833	ABCA1	ATP Binding Cassette Subfamily A Member 1	2.934456348
834	PTHLH	Parathyroid Hormone Like Hormone	2.932133675
835	KMT2E	Lysine Methyltransferase 2E (Inactive)	2.913271904
836	SETD1B	SET Domain Containing 1B, Histone Lysine Methyltransferase	2.913271904
837	ADGRG6	Adhesion G Protein-Coupled Receptor G6	2.907647133
838	SELL	Selectin L	2.899699688
839	CKMT2	Creatine Kinase, Mitochondrial 2	2.893505573
840	MYD88	MYD88 Innate Immune Signal Transduction Adaptor	2.89199543
841	KCNJ10	Potassium Inwardly Rectifying Channel Subfamily J Member 10	2.891505718
842	NR3C2	Nuclear Receptor Subfamily 3 Group C Member 2	2.88309145
843	EDNRB	Endothelin Receptor Type B	2.881774902
844	GLI2	GLI Family Zinc Finger 2	2.881583929
845	DACT1	Dishevelled Binding Antagonist Of Beta Catenin 1	2.873702288
846	SELP	Selectin P	2.858207703
847	IL9	Interleukin 9	2.856477976
848	HSF1	Heat Shock Transcription Factor 1	2.852458477
849	CDC42	Cell Division Cycle 42	2.850028276
850	ADORA2B	Adenosine A2b Receptor	2.816562414
851	NT5C2	5'-Nucleotidase, Cytosolic II	2.814079762
852	IL1R1	Interleukin 1 Receptor Type 1	2.798977852
853	RUNX1	RUNX Family Transcription Factor 1	2.796522141
854	CLDN11	Claudin 11	2.789721727
855	DNAJB1	DnaJ Heat Shock Protein Family (Hsp40) Member B1	2.786218643
856	IL7	Interleukin 7	2.785331011

857	SNCAIP	Synuclein Alpha Interacting Protein	2.781990528
858	ZACN	Zinc Activated Ion Channel	2.78159523
859	LOXL1	Lysyl Oxidase Like 1	2.781576157
860	POLG2	DNA Polymerase Gamma 2, Accessory Subunit	2.77914238
861	CKM	Creatine Kinase, M-Type	2.777067184
862	NR1I2	Nuclear Receptor Subfamily 1 Group I Member 2	2.774758816
863	ZNF142	Zinc Finger Protein 142	2.769249201
864	NES	Nestin	2.76901865
865	CACNA1H	Calcium Voltage-Gated Channel Subunit Alpha1 H	2.754955292
866	AIP	Aryl Hydrocarbon Receptor Interacting Protein	2.748507977
867	IGHE	Immunoglobulin Heavy Constant Epsilon	2.744419813
868	IL22	Interleukin 22	2.744060516
869	CPA6	Carboxypeptidase A6	2.740113497
870	TAGAP	T Cell Activation RhoGTPase Activating Protein	2.739370346
871	GCK	Glucokinase	2.737690926
872	PTPN22	Protein Tyrosine Phosphatase Non-Receptor Type 22	2.729767799
873	SERPINA6	Serpin Family A Member 6	2.72951889
874	ITGAL	Integrin Subunit Alpha L	2.721103191
875	GCG	Glucagon	2.720837593
876	AIRE	Autoimmune Regulator	2.717971325
877	ZFP36	ZFP36 Ring Finger Protein	2.71763587
878	GNB2	G Protein Subunit Beta 2	2.71760869
879	FKBP5	FKBP Prolyl Isomerase 5	2.712097168
880	SELENOI	Selenoprotein I	2.70225215
881	C9orf72	C9orf72-SMCR8 Complex Subunit	2.696703911
882	LPL	Lipoprotein Lipase	2.691449642
883	MBP	Myelin Basic Protein	2.689234018
884	SF1	Splicing Factor 1	2.686408043
885	KL	Klotho	2.685116291
886	C3	Complement C3	2.682346821
887	PSMB9	Proteasome 20S Subunit Beta 9	2.675188065
888	PRKCZ	Protein Kinase C Zeta	2.67309761
889	FBN2	Fibrillin 2	2.672060966
890	CYP27B1	Cytochrome P450 Family 27 Subfamily B Member 1	2.670771837
891	PYY	Peptide YY	2.670077801
892	CKMT1B	Creatine Kinase, Mitochondrial 1B	2.663933277
893	SLPI	Secretory Leukocyte Peptidase Inhibitor	2.661942482
894	IL2RB	Interleukin 2 Receptor Subunit Beta	2.660479069
895	SENP3	SUMO Specific Peptidase 3	2.659738302
896	HAVCR2	Hepatitis A Virus Cellular Receptor 2	2.653107166
897	CD55	CD55 Molecule (Cromer Blood Group)	2.644638538
898	MIR502	MicroRNA 502	2.64247942
899	CLCNKA	Chloride Voltage-Gated Channel Ka	2.641800404

900	IL23A	Interleukin 23 Subunit Alpha	2.641262054
901	TXNRD2	Thioredoxin Reductase 2	2.639935732
902	FCRL3	Fc Receptor Like 3	2.634266376
903	PRKACB	Protein Kinase CAMP-Activated Catalytic Subunit Beta	2.631869078
904	SNORD13	Small Nucleolar RNA, C/D Box 13	2.628620625
905	HTR1B	5-Hydroxytryptamine Receptor 1B	2.62591815
906	AIFM1	Apoptosis Inducing Factor Mitochondria Associated 1	2.62092638
907	GH1	Growth Hormone 1	2.620541096
908	RAB11A	RAB11A, Member RAS Oncogene Family	2.620376587
909	TRPV6	Transient Receptor Potential Cation Channel Subfamily V Member 6	2.616877079
910	PPARGC1A	PPARG Coactivator 1 Alpha	2.614728212
911	NEK1	NIMA Related Kinase 1	2.61028862
912	THRB	Thyroid Hormone Receptor Beta	2.610076666
913	MAOB	Monoamine Oxidase B	2.603140354
914	SHANK3	SH3 And Multiple Ankyrin Repeat Domains 3	2.599887371
915	PFKP	Phosphofructokinase, Platelet	2.593650341
916	TRE-TTC3-1	TRNA-Glu (Anticodon TTC) 3-1	2.589711189
917	SKI	SKI Proto-Oncogene	2.585956573
918	NAV1	Neuron Navigator 1	2.58346653
919	KDM6B	Lysine Demethylase 6B	2.581404924
920	YWHAZ	Tyrosine 3-Monooxygenase/Tryptophan 5-Monooxygenase Activation Protein Zeta	2.576965094
921	BAMBI	BMP And Activin Membrane Bound Inhibitor	2.575575829
922	ANPEP	Alanyl Aminopeptidase, Membrane	2.570040464
923	HLA-DPB1	Major Histocompatibility Complex, Class II, DP Beta 1	2.569588423
924	PTGER3	Prostaglandin E Receptor 3	2.568647623
925	STAT6	Signal Transducer And Activator Of Transcription 6	2.566397905
926	MIR376A1	MicroRNA 376a-1	2.566171646
927	BCHE	Butyrylcholinesterase	2.559315681
928	CHCHD10	Coiled-Coil-Helix-Coiled-Coil-Helix Domain Containing 10	2.557594776
929	CCR3	C-C Motif Chemokine Receptor 3	2.552630424
930	CEBPD	CCAAT Enhancer Binding Protein Delta	2.549837828
931	NUDT6	Nudix Hydrolase 6	2.549812794
932	FLNB	Filamin B	2.547442436
933	VRK1	VRK Serine/Threonine Kinase 1	2.546374798
934	SDHAF2	Succinate Dehydrogenase Complex Assembly Factor 2	2.542903185
935	PPAT	Phosphoribosyl Pyrophosphate Amidotransferase	2.53588438
936	TLR9	Toll Like Receptor 9	2.530705929

937	FOXP2	Forkhead Box P2	2.528961182
938	TBK1	TANK Binding Kinase 1	2.528455019
939	ELK1	ETS Transcription Factor ELK1	2.52751112
940	SPTAN1	Spectrin Alpha, Non-Erythrocytic 1	2.525373936
941	NNAT	Neuronatin	2.524913788
942	CLDN4	Claudin 4	2.522785902
943	CD163	CD163 Molecule	2.519852161
944	AMPD2	Adenosine Monophosphate Deaminase 2	2.516238689
945	SOX5	SRY-Box Transcription Factor 5	2.515477657
946	ASIC2	Acid Sensing Ion Channel Subunit 2	2.512428284
947	DNAJB6	DnaJ Heat Shock Protein Family (Hsp40) Member B6	2.506078005
948	MIR212	MicroRNA 212	2.504137516
949	OTUD6B	OTU Deubiquitinase 6B	2.499084473
950	TBCK	TBC1 Domain Containing Kinase	2.496840715
951	MAPK8IP1	Mitogen-Activated Protein Kinase 8 Interacting Protein 1	2.49572134
952	RRM2B	Ribonucleotide Reductase Regulatory TP53 Inducible Subunit M2B	2.490701675
953	IRS2	Insulin Receptor Substrate 2	2.489195108
954	NEDD4	NEDD4 E3 Ubiquitin Protein Ligase	2.48899436
955	MT-TL1	Mitochondrially Encoded TRNA-Leu (UUA/G) 1	2.485701084
956	GABRA1	Gamma-Aminobutyric Acid Type A Receptor Subunit Alpha1	2.481518269
957	LCAT	Lecithin-Cholesterol Acyltransferase	2.479804993
958	INVS	Inversin	2.479715824
959	SERPINC1	Serpin Family C Member 1	2.473739147
960	P2RY6	Pyrimidinergic Receptor P2Y6	2.468604803
961	RPS27A	Ribosomal Protein S27a	2.467751026
962	SDHAF1	Succinate Dehydrogenase Complex Assembly Factor 1	2.458352566
963	NRXN2	Neurexin 2	2.456090212
964	GHRL	Ghrelin And Obestatin Prepropeptide	2.454140186
965	DCDC2	Doublecortin Domain Containing 2	2.451063633
966	CD28	CD28 Molecule	2.450656891
967	TUBB3	Tubulin Beta 3 Class III	2.449527264
968	CD58	CD58 Molecule	2.446147442
969	SNORD118	Small Nucleolar RNA, C/D Box 118	2.438010454
970	SGK1	Serum/Glucocorticoid Regulated Kinase 1	2.429941177
971	CASC3	CASC3 Exon Junction Complex Subunit	2.423612595
972	PRF1	Perforin 1	2.420913935
973	PPP1R9B	Protein Phosphatase 1 Regulatory Subunit 9B	2.419375896
974	UCN2	Urocortin 2	2.410368204
975	STING1	Stimulator Of Interferon Response CGAMP Interactor 1	2.404247046
976	APOA2	Apolipoprotein A2	2.391481876

977	PRKAR1B	Protein Kinase CAMP-Dependent Type I Regulatory Subunit Beta	2.386893988
978	MED13	Mediator Complex Subunit 13	2.385156155
979	TNFSF11	TNF Superfamily Member 11	2.377963781
980	SETDB1	SET Domain Bifurcated Histone Lysine Methyltransferase 1	2.376712322
981	PPP1R14A	Protein Phosphatase 1 Regulatory Inhibitor Subunit 14A	2.373460293
982	TFAP2A	Transcription Factor AP-2 Alpha	2.373423338
983	SRSF2	Serine And Arginine Rich Splicing Factor 2	2.371556759
984	LNCATV	LncRNA Negative Regulator Of Antiviral Signaling	2.371556759
985	GABRA3	Gamma-Aminobutyric Acid Type A Receptor Subunit Alpha3	2.371300697
986	POLI	DNA Polymerase Iota	2.365101099
987	IL15RA	Interleukin 15 Receptor Subunit Alpha	2.364372253
988	LTBP2	Latent Transforming Growth Factor Beta Binding Protein 2	2.363197803
989	HABP2	Hyaluronan Binding Protein 2	2.358295679
990	TPP1	Tripeptidyl Peptidase 1	2.355720043
991	GPR101	G Protein-Coupled Receptor 101	2.351150036
992	HDAC8	Histone Deacetylase 8	2.349483252
993	DMD	Dystrophin	2.348066568
994	SLC6A2	Solute Carrier Family 6 Member 2	2.344115257
995	TRPC1	Transient Receptor Potential Cation Channel Subfamily C Member 1	2.338437557
996	GFRA1	GDNF Family Receptor Alpha 1	2.336389065
997	SLC9A6	Solute Carrier Family 9 Member A6	2.335343361
998	KMT2B	Lysine Methyltransferase 2B	2.332880974
999	COL17A1	Collagen Type XVII Alpha 1 Chain	2.332007647
1000	TRIM21	Tripartite Motif Containing 21	2.327257156
1001	GLI3	GLI Family Zinc Finger 3	2.326840639
1002	LARGE1	LARGE Xylosyl- And Glucuronyltransferase 1	2.32621789
1003	CDKN3	Cyclin Dependent Kinase Inhibitor 3	2.324233532
1004	IL23R	Interleukin 23 Receptor	2.321604252
1005	POLGARF	POLG Alternative Reading Frame	2.319610596
1006	ERN1	Endoplasmic Reticulum To Nucleus Signaling 1	2.311783314
1007	PFKFB4	6-Phosphofructo-2-Kinase/Fructose-2,6-Biphosphatase 4	2.309811115
1008	UNC13D	Unc-13 Homolog D	2.308903694
1009	MYRIP	Myosin VIIA And Rab Interacting Protein	2.308903694
1010	SYTL3	Synaptotagmin Like 3	2.308903694
1011	SYTL4	Synaptotagmin Like 4	2.308903694
1012	SYTL5	Synaptotagmin Like 5	2.308903694
1013	PTF1A	Pancreas Associated Transcription Factor 1a	2.307240963
1014	CCL3	C-C Motif Chemokine Ligand 3	2.302975178

1015	PKD2	Polycystin 2, Transient Receptor Potential Cation Channel	2.30177784
1016	PLEKHG5	Pleckstrin Homology And RhoGEF Domain Containing G5	2.301665068
1017	HNRNPUL2-BSCL2	HNRNPUL2-BSCL2 Readthrough (NMD Candidate)	2.301665068
1018	UFD1	Ubiquitin Recognition Factor In ER Associated Degradation 1	2.27884388
1019	GLUL	Glutamate-Ammonia Ligase	2.278277636
1020	SLC12A1	Solute Carrier Family 12 Member 1	2.276685715
1021	TRRAP	Transformation/Transcription Domain Associated Protein	2.274324894
1022	MIR323A	MicroRNA 323a	2.273565769
1023	IL10RA	Interleukin 10 Receptor Subunit Alpha	2.267923355
1024	MAPK10	Mitogen-Activated Protein Kinase 10	2.262240171
1025	FAS-AS1	FAS Antisense RNA 1	2.260099411
1026	CYP11B1	Cytochrome P450 Family 11 Subfamily B Member 1	2.254453659
1027	CHRNB4	Cholinergic Receptor Nicotinic Beta 4 Subunit	2.253912926
1028	TNFAIP3	TNF Alpha Induced Protein 3	2.253722906
1029	NONO	Non-POU Domain Containing Octamer Binding	2.252382755
1030	OCA2	OCA2 Melanosomal Transmembrane Protein	2.252380848
1031	MARS1	Methionyl-TRNA Synthetase 1	2.243109703
1032	FREM1	FRAS1 Related Extracellular Matrix 1	2.241195202
1033	MCTS2	MCTS Family Member 2	2.240937948
1034	AFG3L2	AFG3 Like Matrix AAA Peptidase Subunit 2	2.23484993
1035	IQGAP1	IQ Motif Containing GTPase Activating Protein 1	2.234620571
1036	UROD	Uroporphyrinogen Decarboxylase	2.225607395
1037	RRAS2	RAS Related 2	2.202064514
1038	ABCG5	ATP Binding Cassette Subfamily G Member 5	2.201587677
1039	APP	Amyloid Beta Precursor Protein	2.201149464
1040	TMEM127	Transmembrane Protein 127	2.190447569
1041	SERPINF2	Serpin Family F Member 2	2.18864131
1042	NTS	Neurotensin	2.188361645
1043	CSTB	Cystatin B	2.185675144
1044	CCKBR	Cholecystokinin B Receptor	2.185152769
1045	TRPC6	Transient Receptor Potential Cation Channel Subfamily C Member 6	2.184636116
1046	SLC41A1	Solute Carrier Family 41 Member 1	2.180501938
1047	LIPG	Lipase G, Endothelial Type	2.177545309
1048	HAVCR1	Hepatitis A Virus Cellular Receptor 1	2.174080372
1049	UFC1	Ubiquitin-Fold Modifier Conjugating Enzyme 1	2.172990084
1050	TGM2	Transglutaminase 2	2.171761751

1051	CRHR2	Corticotropin Releasing Hormone Receptor 2	2.168218613
1052	TPO	Thyroid Peroxidase	2.165794849
1053	SVIP	Small VCP Interacting Protein	2.165766239
1054	KEAP1	Kelch Like ECH Associated Protein 1	2.163036585
1055	KLRK1	Killer Cell Lectin Like Receptor K1	2.162946463
1056	TK1	Thymidine Kinase 1	2.15841651
1057	MEST	Mesoderm Specific Transcript	2.154789448
1058	GABRB3	Gamma-Aminobutyric Acid Type A Receptor Subunit Beta3	2.153085232
1059	TAAR1	Trace Amine Associated Receptor 1	2.15085125
1060	DPP4	Dipeptidyl Peptidase 4	2.14468503
1061	GAP43	Growth Associated Protein 43	2.14283061
1062	ADRB1	Adrenoceptor Beta 1	2.138268948
1063	GZMB	Granzyme B	2.134093761
1064	TAP1	Transporter 1, ATP Binding Cassette Subfamily B Member	2.12492609
1065	SSTR5	Somatostatin Receptor 5	2.121477127
1066	LCK	LCK Proto-Oncogene, Src Family Tyrosine Kinase	2.11997509
1067	SLC5A2	Solute Carrier Family 5 Member 2	2.118101358
1068	PPT1	Palmitoyl-Protein Thioesterase 1	2.117379665
1069	TTN	Titin	2.115671158
1070	FOXE1	Forkhead Box E1	2.110120296
1071	ALDH7A1	Aldehyde Dehydrogenase 7 Family Member A1	2.108501196
1072	PRKACG	Protein Kinase CAMP-Activated Catalytic Subunit Gamma	2.106798649
1073	FAAH	Fatty Acid Amide Hydrolase	2.103232622
1074	OCLN	Occludin	2.102009296
1075	CALB2	Calbindin 2	2.098790646
1076	BLVRB	Biliverdin Reductase B	2.093677998
1077	PCDH10	Protocadherin 10	2.086743355
1078	GRK2	G Protein-Coupled Receptor Kinase 2	2.084269762
1079	SYNGAP1	Synaptic Ras GTPase Activating Protein 1	2.080559969
1080	PRPF8	Pre-MRNA Processing Factor 8	2.080421686
1081	GATA1	GATA Binding Protein 1	2.07137537
1082	NCL	Nucleolin	2.068956852
1083	HTR1D	5-Hydroxytryptamine Receptor 1D	2.067359686
1084	WAS	WASP Actin Nucleation Promoting Factor	2.064549208
1085	GHRH	Growth Hormone Releasing Hormone	2.0631814
1086	UBASH3A	Ubiquitin Associated And SH3 Domain Containing A	2.0629704
1087	DNM1L	Dynamin 1 Like	2.060434341
1088	MIR181C	MicroRNA 181c	2.046938419
1089	KCNN1	Potassium Calcium-Activated Channel Subfamily N Member 1	2.040838718
1090	PRPSAP1	Phosphoribosyl Pyrophosphate Synthetase Associated Protein 1	2.037351608

1091	PGAP1	Post-GPI Attachment To Proteins Inositol Deacylase 1	2.036619186
1092	ANKK1	Ankyrin Repeat And Kinase Domain Containing 1	2.03550005
1093	IL16	Interleukin 16	2.033810854
1094	MEF2D	Myocyte Enhancer Factor 2D	2.029028177
1095	MAPK7	Mitogen-Activated Protein Kinase 7	2.028534412
1096	CS	Citrate Synthase	2.027335167
1097	GABBR1	Gamma-Aminobutyric Acid Type B Receptor Subunit 1	2.026267529
1098	MFGE8	Milk Fat Globule EGF And Factor V/VIII Domain Containing	2.024183273
1099	MIR7-3HG	MIR7-3 Host Gene	2.01949358
1100	ERVW-1	Endogenous Retrovirus Group W Member 1, Envelope	2.018024445
1101	CYP21A2	Cytochrome P450 Family 21 Subfamily A Member 2	2.017075062
1102	FYN	FYN Proto-Oncogene, Src Family Tyrosine Kinase	2.009876013
1103	SPRY1	Sprouty RTK Signaling Antagonist 1	2.00982976
1104	RTN4	Reticulon 4	2.000619411
1105	TBX21	T-Box Transcription Factor 21	1.99963522
1106	ASIC1	Acid Sensing Ion Channel Subunit 1	1.999064684
1107	SORBS1	Sorbin And SH3 Domain Containing 1	1.998641253
1108	PTPN1	Protein Tyrosine Phosphatase Non-Receptor Type 1	1.99827683
1109	CRY1	Cryptochrome Circadian Regulator 1	1.996465206
1110	ADORA2A	Adenosine A2a Receptor	1.990542889
1111	PNPT1	Polyribonucleotide Nucleotidyltransferase 1	1.984317541
1112	MED27	Mediator Complex Subunit 27	1.984317541
1113	INTS5	Integrator Complex Subunit 5	1.98187077
1114	MT-CO1	Mitochondrially Encoded Cytochrome C Oxidase I	1.980407953
1115	PDX1	Pancreatic And Duodenal Homeobox 1	1.979709744
1116	CAV3	Caveolin 3	1.973586202
1117	CYP7A1	Cytochrome P450 Family 7 Subfamily A Member 1	1.96939826
1118	CASP1	Caspase 1	1.961028934
1119	BMPR2	Bone Morphogenetic Protein Receptor Type 2	1.95752871
1120	LBR	Lamin B Receptor	1.95751667
1121	KCNB2	Potassium Voltage-Gated Channel Subfamily B Member 2	1.95378685
1122	FBL	Fibrillarin	1.953006387
1123	SCNN1B	Sodium Channel Epithelial 1 Subunit Beta	1.952883124
1124	SCNN1G	Sodium Channel Epithelial 1 Subunit Gamma	1.952883124
1125	SLCO1B1	Solute Carrier Organic Anion Transporter Family Member 1B1	1.937621355

1126	ABCC9	ATP Binding Cassette Subfamily C Member 9	1.936583519
1127	PAFAH1B1	Platelet Activating Factor Acetylhydrolase 1b Regulatory Subunit 1	1.933475256
1128	CBX2	Chromobox 2	1.93292141
1129	PRKAA2	Protein Kinase AMP-Activated Catalytic Subunit Alpha 2	1.923704743
1130	PVALB	Parvalbumin	1.91356051
1131	UBE3A	Ubiquitin Protein Ligase E3A	1.904678345
1132	GP1BB	Glycoprotein Ib Platelet Subunit Beta	1.899169326
1133	HIRA	Histone Cell Cycle Regulator	1.899169326
1134	SEC24C	SEC24 Homolog C, COPII Coat Complex Component	1.899169326
1135	JMJD1C	Jumonji Domain Containing 1C	1.899169326
1136	SLC1A2	Solute Carrier Family 1 Member 2	1.89783597
1137	SV2C	Synaptic Vesicle Glycoprotein 2C	1.896938086
1138	OCRL	OCRL Inositol Polyphosphate-5-Phosphatase	1.89579308
1139	IL12A	Interleukin 12A	1.893318534
1140	PFKFB3	6-Phosphofructo-2-Kinase/Fructose-2,6-Biphosphatase 3	1.892617106
1141	C4A	Complement C4A (Chido/Rodgers Blood Group)	1.890674233
1142	IKBKB	Inhibitor Of Nuclear Factor Kappa B Kinase Subunit Beta	1.889453411
1143	AHSG	Alpha 2-HS Glycoprotein	1.887336493
1144	ADCY10	Adenylate Cyclase 10	1.887128592
1145	CETP	Cholesteryl Ester Transfer Protein	1.881660223
1146	LIPC	Lipase C, Hepatic Type	1.881660223
1147	CHRNA2	Cholinergic Receptor Nicotinic Alpha 2 Subunit	1.881199956
1148	GABRG2	Gamma-Aminobutyric Acid Type A Receptor Subunit Gamma2	1.881199956
1149	CHRNB2	Cholinergic Receptor Nicotinic Beta 2 Subunit	1.881199956
1150	ADAM17	ADAM Metallopeptidase Domain 17	1.877621055
1151	ATP4A	ATPase H+/K+ Transporting Subunit Alpha	1.875597239
1152	TRAPPC14	Trafficking Protein Particle Complex Subunit 14	1.872573376
1153	ACP5	Acid Phosphatase 5, Tartrate Resistant	1.865718007
1154	CD69	CD69 Molecule	1.864874721
1155	FURIN	Furin, Paired Basic Amino Acid Cleaving Enzyme	1.856804967
1156	FTL	Ferritin Light Chain	1.853549719
1157	ATF6B	Activating Transcription Factor 6 Beta	1.85325861
1158	GRIN1	Glutamate Ionotropic Receptor NMDA Type Subunit 1	1.84885478
1159	PNMT	Phenylethanolamine N-Methyltransferase	1.846174359
1160	BICD2	BICD Cargo Adaptor 2	1.845841408

1161	APOC3	Apolipoprotein C3	1.843976378
1162	RPGRIP1	RPGR Interacting Protein 1	1.840808988
1163	RIGI	RNA Sensor RIG-I	1.83446157
1164	CYFIP1	Cytoplasmic FMR1 Interacting Protein 1	1.830850363
1165	AGER	Advanced Glycosylation End-Product Specific Receptor	1.827825189
1166	IER3IP1	Immediate Early Response 3 Interacting Protein 1	1.826164842
1167	DLG4	Discs Large MAGUK Scaffold Protein 4	1.825685859
1168	DDIT3	DNA Damage Inducible Transcript 3	1.823389411
1169	MATN3	Matrilin 3	1.819538474
1170	SPRY2	Sprouty RTK Signaling Antagonist 2	1.80561173
1171	DOCK3	Dedicator Of Cytokinesis 3	1.802378178
1172	STRA6	Signaling Receptor And Transporter Of Retinol STRA6	1.801985621
1173	SLCO1C1	Solute Carrier Organic Anion Transporter Family Member 1C1	1.801813602
1174	POGZ	Pogo Transposable Element Derived With ZNF Domain	1.799245596
1175	CCL4	C-C Motif Chemokine Ligand 4	1.793910861
1176	PLXNA1	Plexin A1	1.791612983
1177	CHD8	Chromodomain Helicase DNA Binding Protein 8	1.79046917
1178	ADAM10	ADAM Metallopeptidase Domain 10	1.788002729
1179	MAP3K1	Mitogen-Activated Protein Kinase Kinase Kinase 1	1.786929488
1180	CIITA	Class II Major Histocompatibility Complex Transactivator	1.786873817
1181	GCH1	GTP Cyclohydrolase 1	1.780113578
1182	PRTN3	Proteinase 3	1.778058529
1183	PCBP2	Poly(RC) Binding Protein 2	1.761238217
1184	CLASRP	CLK4 Associating Serine/Arginine Rich Protein	1.760467291
1185	DAXX	Death Domain Associated Protein	1.755101562
1186	CALB1	Calbindin 1	1.754067898
1187	SUMF1	Sulfatase Modifying Factor 1	1.752137899
1188	CD1D	CD1d Molecule	1.751890659
1189	SEMA3A	Semaphorin 3A	1.746395588
1190	DPM1	Dolichyl-Phosphate Mannosyltransferase Subunit 1, Catalytic	1.745368361
1191	MAX	MYC Associated Factor X	1.745097995
1192	HES1	Hes Family BHLH Transcription Factor 1	1.74189496
1193	CNGB1	Cyclic Nucleotide Gated Channel Subunit Beta 1	1.738204956
1194	FCGR3A	Fc Gamma Receptor IIIa	1.73728776
1195	RAB17	RAB17, Member RAS Oncogene Family	1.736718178
1196	SLC25A4	Solute Carrier Family 25 Member 4	1.73533988
1197	HTT	Huntingtin	1.734892368
1198	RETN	Resistin	1.731728911

1199	TREX1	Three Prime Repair Exonuclease 1	1.725084424
1200	CYP26C1	Cytochrome P450 Family 26 Subfamily C Member 1	1.721565485
1201	AVPR1B	Arginine Vasopressin Receptor 1B	1.715523481
1202	NLGN2	Neuroligin 2	1.707640409
1203	TTC12	Tetratricopeptide Repeat Domain 12	1.707640409
1204	P2RX5-TAX1BP3	P2RX5-TAX1BP3 Readthrough (NMD Candidate)	1.705595374
1205	RBFOX3	RNA Binding Fox-1 Homolog 3	1.704753041
1206	TFEB	Transcription Factor EB	1.703433514
1207	CAPN2	Calpain 2	1.699089527
1208	ATG16L1	Autophagy Related 16 Like 1	1.686106086
1209	MIR155HG	MIR155 Host Gene	1.685986996
1210	ADAMTS1	ADAM Metallopeptidase With Thrombospondin Type 1 Motif 1	1.685710907
1211	GRM5	Glutamate Metabotropic Receptor 5	1.685474873
1212	SLBP	Stem-Loop Histone MRNA Binding Protein	1.683817267
1213	MC2R	Melanocortin 2 Receptor	1.682412386
1214	CR2	Complement C3d Receptor 2	1.678302646
1215	RAB33A	RAB33A, Member RAS Oncogene Family	1.677010417
1216	ARG1	Arginase 1	1.676432729
1217	GRK4	G Protein-Coupled Receptor Kinase 4	1.67594111
1218	RRM2	Ribonucleotide Reductase Regulatory Subunit M2	1.674868941
1219	TAP2	Transporter 2, ATP Binding Cassette Subfamily B Member	1.666711926
1220	HINT1	Histidine Triad Nucleotide Binding Protein 1	1.658688903
1221	MAF	MAF BZIP Transcription Factor	1.651806831
1222	SYT1	Synaptotagmin 1	1.648489952
1223	SULF1	Sulfatase 1	1.645891786
1224	KCNA1	Potassium Voltage-Gated Channel Subfamily A Member 1	1.640309691
1225	IL33	Interleukin 33	1.639780283
1226	CLDN16	Claudin 16	1.639344931
1227	LRBA	LPS Responsive Beige-Like Anchor Protein	1.635534286
1228	SOX10	SRY-Box Transcription Factor 10	1.632872224
1229	NRXN1	Neurexin 1	1.629869342
1230	EMP2	Epithelial Membrane Protein 2	1.620692372
1231	PGF	Placental Growth Factor	1.618308067
1232	PTPN13	Protein Tyrosine Phosphatase Non-Receptor Type 13	1.617014647
1233	CYP11A1	Cytochrome P450 Family 11 Subfamily A Member 1	1.61619556
1234	KLRC1	Killer Cell Lectin Like Receptor C1	1.61592257
1235	MYO1C	Myosin IC	1.607628703
1236	FHL2	Four And A Half LIM Domains 2	1.606284499

1237	GHRHR	Growth Hormone Releasing Hormone Receptor	1.605847716
1238	PPP1R1B	Protein Phosphatase 1 Regulatory Inhibitor Subunit 1B	1.604619026
1239	TNFSF13B	TNF Superfamily Member 13b	1.600982428
1240	GDF11	Growth Differentiation Factor 11	1.600921988
1241	IMPDH2	Inosine Monophosphate Dehydrogenase 2	1.596850038
1242	ATP12A	ATPase H+/K+ Transporting Non-Gastric Alpha2 Subunit	1.591700912
1243	FABP2	Fatty Acid Binding Protein 2	1.58516562
1244	CLCN2	Chloride Voltage-Gated Channel 2	1.578976512
1245	FLT3	Fms Related Receptor Tyrosine Kinase 3	1.575453997
1246	ANO10	Anoctamin 10	1.566387892
1247	ADRA1B	Adrenoceptor Alpha 1B	1.565168858
1248	PDYN	Prodynorphin	1.561677456
1249	DDC	Dopa Decarboxylase	1.556981325
1250	PRKAA1	Protein Kinase AMP-Activated Catalytic Subunit Alpha 1	1.554337382
1251	MLC1	Modulator Of VRAC Current 1	1.553800464
1252	SOS1	SOS Ras/Rac Guanine Nucleotide Exchange Factor 1	1.552295208
1253	GRK3	G Protein-Coupled Receptor Kinase 3	1.550262332
1254	CYP2B6	Cytochrome P450 Family 2 Subfamily B Member 6	1.549967527
1255	ABCB4	ATP Binding Cassette Subfamily B Member 4	1.549215436
1256	ITCH	Itchy E3 Ubiquitin Protein Ligase	1.548682332
1257	MT-ATP6	Mitochondrially Encoded ATP Synthase Membrane Subunit 6	1.538587093
1258	NOX1	NADPH Oxidase 1	1.538255334
1259	APOC2	Apolipoprotein C2	1.53307426
1260	COL2A1	Collagen Type II Alpha 1 Chain	1.531146884
1261	SAG	S-Antigen Visual Arrestin	1.527583122
1262	MSL3	MSL Complex Subunit 3	1.526978731
1263	GRM1	Glutamate Metabotropic Receptor 1	1.523578048
1264	CD19	CD19 Molecule	1.521991849
1265	LTC4S	Leukotriene C4 Synthase	1.521199226
1266	TNFRSF13B	TNF Receptor Superfamily Member 13B	1.52051127
1267	BSND	Barttin CLCNK Type Accessory Subunit Beta	1.507877588
1268	MBTPS1	Membrane Bound Transcription Factor Peptidase, Site 1	1.506035686
1269	UBQLN2	Ubiquilin 2	1.505466223
1270	ADORA1	Adenosine A1 Receptor	1.504671454
1271	RBL1	RB Transcriptional Corepressor Like 1	1.498908162
1272	IFNB1	Interferon Beta 1	1.496151209
1273	SOX14	SRY-Box Transcription Factor 14	1.492606759

1274	MT-ND5	Mitochondrially Encoded NADH:Ubiquinone Oxidoreductase Core Subunit 5	1.489795804
1275	CYP2C8	Cytochrome P450 Family 2 Subfamily C Member 8	1.486140132
1276	LYN	LYN Proto-Oncogene, Src Family Tyrosine Kinase	1.485756636
1277	ATP2B3	ATPase Plasma Membrane Ca2+ Transporting 3	1.481213927
1278	HPS6	HPS6 Biogenesis Of Lysosomal Organelles Complex 2 Subunit 3	1.480613351
1279	YWHAQ	Tyrosine 3-Monooxygenase/Tryptophan 5-Monooxygenase Activation Protein Theta	1.480381489
1280	PABPC1	Poly(A) Binding Protein Cytoplasmic 1	1.475040674
1281	LIN7C	Lin-7 Homolog C, Crumbs Cell Polarity Complex Component	1.471921444
1282	GAD1	Glutamate Decarboxylase 1	1.469410658
1283	SRSF7	Serine And Arginine Rich Splicing Factor 7	1.467658043
1284	GPBAR1	G Protein-Coupled Bile Acid Receptor 1	1.462593317
1285	G6PC1	Glucose-6-Phosphatase Catalytic Subunit 1	1.460130334
1286	MAP2	Microtubule Associated Protein 2	1.454602122
1287	AKAP1	A-Kinase Anchoring Protein 1	1.453994751
1288	SNRPD1	Small Nuclear Ribonucleoprotein D1 Polypeptide	1.4507792
1289	FABP3	Fatty Acid Binding Protein 3	1.448719144
1290	SPRED1	Sprouty Related EVH1 Domain Containing 1	1.440581322
1291	DRD3	Dopamine Receptor D3	1.440576077
1292	CLN6	CLN6 Transmembrane ER Protein	1.440323353
1293	DSG3	Desmoglein 3	1.440074325
1294	SRSF1	Serine And Arginine Rich Splicing Factor 1	1.438736558
1295	ABCC5	ATP Binding Cassette Subfamily C Member 5	1.437035203
1296	SLC2A9	Solute Carrier Family 2 Member 9	1.433818817
1297	TRAF2	TNF Receptor Associated Factor 2	1.430304289
1298	RHO	Rhodopsin	1.427010059
1299	HPX	Hemopexin	1.424868345
1300	AUTS2	Activator Of Transcription And Developmental Regulator AUTS2	1.423401833
1301	HBB	Hemoglobin Subunit Beta	1.419036627
1302	RPS6	Ribosomal Protein S6	1.407962203
1303	GRB10	Growth Factor Receptor Bound Protein 10	1.404852271
1304	LOC654780	Splicing Factor Proline/Glutamine-Rich	1.403206944
1305	CNN1	Calponin 1	1.398741245
1306	SLC52A2	Solute Carrier Family 52 Member 2	1.395714164
1307	GABBR2	Gamma-Aminobutyric Acid Type B Receptor Subunit 2	1.392204881

1308	HCN1	Hyperpolarization Activated Cyclic Nucleotide Gated Potassium Channel 1	1.392204881
1309	HCN2	Hyperpolarization Activated Cyclic Nucleotide Gated Potassium And Sodium Channel 2	1.392204881
1310	IL17RB	Interleukin 17 Receptor B	1.392204881
1311	CCDC6	Coiled-Coil Domain Containing 6	1.392204881
1312	UROS	Uroporphyrinogen III Synthase	1.389525652
1313	NIPA2	NIPA Magnesium Transporter 2	1.384832144
1314	KLK5	Kallikrein Related Peptidase 5	1.379300952
1315	MYO5B	Myosin VB	1.376662016
1316	ETHE1	ETHE1 Persulfide Dioxygenase	1.376330256
1317	NEDD4L	NEDD4 Like E3 Ubiquitin Protein Ligase	1.374375582
1318	CD5	CD5 Molecule	1.371708274
1319	CDK8	Cyclin Dependent Kinase 8	1.367568374
1320	DHRS9	Dehydrogenase/Reductase 9	1.36679101
1321	STAT4	Signal Transducer And Activator Of Transcription 4	1.357135057
1322	HLA-DRA	Major Histocompatibility Complex, Class II, DR Alpha	1.357135057
1323	LAMA2	Laminin Subunit Alpha 2	1.356424332
1324	FGR	FGR Proto-Oncogene, Src Family Tyrosine Kinase	1.355626106
1325	SLC1A3	Solute Carrier Family 1 Member 3	1.35157752
1326	CILK1	Ciliogenesis Associated Kinase 1	1.350384474
1327	P2RX5	Purinergic Receptor P2X 5	1.348006368
1328	GALR1	Galanin Receptor 1	1.345736742
1329	CDKL5	Cyclin Dependent Kinase Like 5	1.343001604
1330	DHX58	DExH-Box Helicase 58	1.341890693
1331	RBM23	RNA Binding Motif Protein 23	1.341890693
1332	MICA	MHC Class I Polypeptide-Related Sequence A	1.339882135
1333	CFB	Complement Factor B	1.336983323
1334	MZF1	Myeloid Zinc Finger 1	1.336772561
1335	H3-4	H3.4 Histone, Cluster Member	1.326677322
1336	NEUROD1	Neuronal Differentiation 1	1.326653004
1337	PADI4	Peptidyl Arginine Deiminase 4	1.323977113
1338	TRAF3	TNF Receptor Associated Factor 3	1.323334098
1339	SLC11A2	Solute Carrier Family 11 Member 2	1.322047472
1340	PEG10	Paternally Expressed 10	1.318081498
1341	LALBA	Lactalbumin Alpha	1.318081498
1342	RNU4ATAC	RNA, U4atac Small Nuclear	1.316115856
1343	PTPN6	Protein Tyrosine Phosphatase Non-Receptor Type 6	1.307402015
1344	SLC12A5	Solute Carrier Family 12 Member 5	1.298190594
1345	TRAF1	TNF Receptor Associated Factor 1	1.298190594
1346	RAB3GAP2	RAB3 GTPase Activating Non-Catalytic Protein Subunit 2	1.297335625
1347	ABCB10	ATP Binding Cassette Subfamily B Member 10	1.296157122

1348	CNTNAP2	Contactin Associated Protein 2	1.296091795
1349	OXTR	Oxytocin Receptor	1.291180134
1350	FBLN2	Fibulin 2	1.29049325
1351	MICU1	Mitochondrial Calcium Uptake 1	1.28959167
1352	PRPS2	Phosphoribosyl Pyrophosphate Synthetase 2	1.288080573
1353	PRPS1L1	Phosphoribosyl Pyrophosphate Synthetase 1 Like 1	1.288080573
1354	CPT2	Carnitine Palmitoyltransferase 2	1.280269861
1355	SLC12A2	Solute Carrier Family 12 Member 2	1.278155088
1356	GYPC	Glycophorin C (Gerbich Blood Group)	1.277323365
1357	SCN11A	Sodium Voltage-Gated Channel Alpha Subunit 11	1.267165065
1358	TUBGCP5	Tubulin Gamma Complex Component 5	1.266786575
1359	PPY	Pancreatic Polypeptide	1.265613437
1360	SCN5A	Sodium Voltage-Gated Channel Alpha Subunit 5	1.258544922
1361	PVR	PVR Cell Adhesion Molecule	1.25084126
1362	SGCE	Sarcoglycan Epsilon	1.240377426
1363	NFKBIB	NFKB Inhibitor Beta	1.238869429
1364	PTPRK	Protein Tyrosine Phosphatase Receptor Type K	1.238621354
1365	SLC18A2	Solute Carrier Family 18 Member A2	1.230490446
1366	SCN1B	Sodium Voltage-Gated Channel Beta Subunit 1	1.225395083
1367	HLA-DPA1	Major Histocompatibility Complex, Class II, DP Alpha 1	1.225395083
1368	PRRT2	Proline Rich Transmembrane Protein 2	1.224621892
1369	IL18R1	Interleukin 18 Receptor 1	1.222670078
1370	S1PR2	Sphingosine-1-Phosphate Receptor 2	1.220957875
1371	IL27	Interleukin 27	1.220957875
1372	MYT1L	Myelin Transcription Factor 1 Like	1.217674017
1373	DIRAS2	DIRAS Family GTPase 2	1.217674017
1374	CLCN1	Chloride Voltage-Gated Channel 1	1.207484126
1375	INA	Internexin Neuronal Intermediate Filament Protein Alpha	1.207484126
1376	EBI3	Epstein-Barr Virus Induced 3	1.207484126
1377	HLA-C	Major Histocompatibility Complex, Class I, C	1.206570029
1378	CSAD	Cysteine Sulfinic Acid Decarboxylase	1.206570029
1379	TTC7A	Tetratricopeptide Repeat Domain 7A	1.204907775
1380	R3HDM2	R3H Domain Containing 2	1.201269865
1381	CARMIL1	Capping Protein Regulator And Myosin 1 Linker 1	1.201269865
1382	PRPSAP2	Phosphoribosyl Pyrophosphate Synthetase Associated Protein 2	1.201269865
1383	B3GNT4	UDP-GlcNAc:BetaGal Beta-1,3-N-Acetylglucosaminyltransferase 4	1.201269865
1384	TMEM171	Transmembrane Protein 171	1.201269865
1385	AQP5	Aquaporin 5	1.200212121

1386	FCGR2B	Fc Gamma Receptor IIb	1.193787336
1387	SSBP2	Single Stranded DNA Binding Protein 2	1.19116652
1388	TLR3	Toll Like Receptor 3	1.186131477
1389	CNTF	Ciliary Neurotrophic Factor	1.186131477
1390	GRM8	Glutamate Metabotropic Receptor 8	1.184887648
1391	SLC5A1	Solute Carrier Family 5 Member 1	1.182165623
1392	ORAI1	ORAI Calcium Release-Activated Calcium Modulator 1	1.181343675
1393	NR1I3	Nuclear Receptor Subfamily 1 Group I Member 3	1.181191683
1394	KLF10	KLF Transcription Factor 10	1.18029511
1395	PENK	Proenkephalin	1.18029511
1396	VTRNA1-1	Vault RNA 1-1	1.18029511
1397	PSPN	Persephin	1.180228949
1398	CLEC16A	C-Type Lectin Domain Containing 16A	1.179975986
1399	AVPR1A	Arginine Vasopressin Receptor 1A	1.178046227
1400	TPH1	Tryptophan Hydroxylase 1	1.178046227
1401	FXR2	FMR1 Autosomal Homolog 2	1.178046227
1402	NOTCH4	Notch Receptor 4	1.173037291
1403	TRPM4	Transient Receptor Potential Cation Channel Subfamily M Member 4	1.172235727
1404	USF1	Upstream Transcription Factor 1	1.157766938
1405	HEXA	Hexosaminidase Subunit Alpha	1.155085325
1406	KLRB1	Killer Cell Lectin Like Receptor B1	1.151955485
1407	FBN3	Fibrillin 3	1.150750279
1408	DAB1	DAB Adaptor Protein 1	1.147726536
1409	HRH3	Histamine Receptor H3	1.141973615
1410	GLIS3	GLIS Family Zinc Finger 3	1.141630411
1411	CD247	CD247 Molecule	1.138992548
1412	AMPH	Amphiphysin	1.137340188
1413	ENPEP	Glutamyl Aminopeptidase	1.133269072
1414	SHOX2	SHOX Homeobox 2	1.133269072
1415	RBFOX1	RNA Binding Fox-1 Homolog 1	1.130485058
1416	ITGAM	Integrin Subunit Alpha M	1.125546575
1417	PITRM1-AS1	PITRM1 Antisense RNA 1	1.124916553
1418	IRF4	Interferon Regulatory Factor 4	1.120799422
1419	TRAF5	TNF Receptor Associated Factor 5	1.118472457
1420	RELN	Reelin	1.116361499
1421	SCN2A	Sodium Voltage-Gated Channel Alpha Subunit 2	1.114085436
1422	SCT	Secretin	1.110529661
1423	DGKQ	Diacylglycerol Kinase Theta	1.110292077
1424	NEFH	Neurofilament Heavy Chain	1.110121131
1425	RAB5A	RAB5A, Member RAS Oncogene Family	1.110121131
1426	PLTP	Phospholipid Transfer Protein	1.105235219
1427	FCGRT	Fc Gamma Receptor And Transporter	1.105235219
1428	RPL28	Ribosomal Protein L28	1.105235219
1429	SLC38A2	Solute Carrier Family 38 Member 2	1.105235219
1430	LYRM4	LYR Motif Containing 4	1.097085953
1431	REL	REL Proto-Oncogene, NF-KB Subunit	1.096212626

1432	CHRFAM7A	CHRNA7 (Exons 5-10) And FAM7A (Exons A-E) Fusion	1.096212626
1433	TNFRSF8	TNF Receptor Superfamily Member 8	1.094961405
1434	SLC19A2	Solute Carrier Family 19 Member 2	1.094034433
1435	AGO2	Argonaute RISC Catalytic Component 2	1.093763113
1436	CLOCK	Clock Circadian Regulator	1.091553926
1437	ST3GAL5	ST3 Beta-Galactoside Alpha-2,3-Sialyl-transferase 5	1.088813543
1438	MAGI2	Membrane Associated Guanylate Kinase, WW And PDZ Domain Containing 2	1.081708908
1439	KLK10	Kallikrein Related Peptidase 10	1.078305006
1440	LRP1	LDL Receptor Related Protein 1	1.077535748
1441	SCG2	Secretogranin II	1.075414777
1442	KCNS3	Potassium Voltage-Gated Channel Modifier Subfamily S Member 3	1.075414777
1443	VAX2	Ventral Anterior Homeobox 2	1.075414777
1444	USH1C	USH1 Protein Network Component Harmonin	1.07067585
1445	IL17F	Interleukin 17F	1.065912247
1446	PRKCQ	Protein Kinase C Theta	1.06383419
1447	GOSR2	Golgi SNAP Receptor Complex Member 2	1.06383419
1448	DSC1	Desmocollin 1	1.058278441
1449	MIR615	MicroRNA 615	1.05744946
1450	IFIH1	Interferon Induced With Helicase C Domain 1	1.057158947
1451	EFS	Embryonal Fyn-Associated Substrate	1.054831862
1452	ARTN	Artemin	1.054060221
1453	WDR48	WD Repeat Domain 48	1.054060221
1454	DLX5	Distal-Less Homeobox 5	1.051380634
1455	CACNB2	Calcium Voltage-Gated Channel Auxiliary Subunit Beta 2	1.049786091
1456	PDGFA	Platelet Derived Growth Factor Subunit A	1.043839455
1457	DCHS1	Dachsous Cadherin-Related 1	1.042229772
1458	PDPK1	3-Phosphoinositide Dependent Protein Kinase 1	1.040216684
1459	PNP	Purine Nucleoside Phosphorylase	1.039519668
1460	ICAM2	Intercellular Adhesion Molecule 2	1.038931489
1461	INPP4B	Inositol Polyphosphate-4-Phosphatase Type II B	1.038931489
1462	CGAS	Cyclic GMP-AMP Synthase	1.038931489
1463	CHRNA5	Cholinergic Receptor Nicotinic Alpha 5 Subunit	1.035507917
1464	KDM5C	Lysine Demethylase 5C	1.035336018
1465	KCNA2	Potassium Voltage-Gated Channel Subfamily A Member 2	1.027788401
1466	TNFRSF13C	TNF Receptor Superfamily Member 13C	1.026136041
1467	SMN1	Survival Of Motor Neuron 1, Telomeric	1.026136041

1468	SMN2	Survival Of Motor Neuron 2, Centromeric	1.026136041
1469	ADSL	Adenylosuccinate Lyase	1.0242064
1470	DCTN1	Dynactin Subunit 1	1.0242064
1471	PHOX2A	Paired Like Homeobox 2A	1.0242064
1472	EIF2B5	Eukaryotic Translation Initiation Factor 2B Subunit Epsilon	1.0242064
1473	LOC102724058	Uncharacterized LOC102724058	1.0242064
1474	XPR1	Xenotropic And Polytropic Retrovirus Receptor 1	1.024161577
1475	POLR2L	RNA Polymerase II, I And III Subunit L	1.02296257
1476	NLRP1	NLR Family Pyrin Domain Containing 1	1.021898985
1477	SLC2A2	Solute Carrier Family 2 Member 2	1.02169919
1478	MTTP	Microsomal Triglyceride Transfer Protein	1.02169919
1479	ABCG1	ATP Binding Cassette Subfamily G Member 1	1.02169919
1480	ASPH	Aspartate Beta-Hydroxylase	1.017364025
1481	GRM7	Glutamate Metabotropic Receptor 7	1.014568567
1482	SLC32A1	Solute Carrier Family 32 Member 1	1.014568567
1483	DLK1	Delta Like Non-Canonical Notch Ligand 1	1.013127565
1484	DSG1	Desmoglein 1	1.010732412
1485	CELF5	CUGBP Elav-Like Family Member 5	1.00795424
1486	ASTN2	Astrotactin 2	1.001510143
1487	DYNC1H1	Dynein Cytoplasmic 1 Heavy Chain 1	0.996987283
1488	MT-ND4	Mitochondrially Encoded NADH:Ubiquinone Oxidoreductase Core Subunit 4	0.996987283
1489	ZNF335	Zinc Finger Protein 335	0.99138546
1490	VGLL4	Vestigial Like Family Member 4	0.99138546
1491	TPH2	Tryptophan Hydroxylase 2	0.982575953
1492	GRIK5	Glutamate Ionotropic Receptor Kainate Type Subunit 5	0.982575953
1493	PDLIM7	PDZ And LIM Domain 7	0.981877506
1494	COX4I1	Cytochrome C Oxidase Subunit 4I1	0.980765164
1495	CLDN19	Claudin 19	0.978300214
1496	PTPN2	Protein Tyrosine Phosphatase Non-Receptor Type 2	0.968900621
1497	GRM4	Glutamate Metabotropic Receptor 4	0.966356516
1498	MC4R	Melanocortin 4 Receptor	0.953765154
1499	LINC01554	Long Intergenic Non-Protein Coding RNA 1554	0.95194912
1500	NEUROG3	Neurogenin 3	0.94941467
1501	SCN4A	Sodium Voltage-Gated Channel Alpha Subunit 4	0.947270036
1502	EPM2A	EPM2A Glucan Phosphatase, Laforin	0.945492744
1503	NHLRC1	NHL Repeat Containing E3 Ubiquitin Protein Ligase 1	0.945492744
1504	GPRC6A	G Protein-Coupled Receptor Class C Group 6 Member A	0.943522334

1505	ADK	Adenosine Kinase	0.94237268
1506	SBDS	SBDS Ribosome Maturation Factor	0.94237268
1507	UCN3	Urocortin 3	0.940045714
1508	CHRNA7	Cholinergic Receptor Nicotinic Alpha 7 Subunit	0.937869608
1509	MAN2A1	Mannosidase Alpha Class 2A Member 1	0.9377141
1510	PREP	Prolyl Endopeptidase	0.9377141
1511	IARS1	Isoleucyl-TRNA Synthetase 1	0.93177855
1512	KIF21A	Kinesin Family Member 21A	0.925430417
1513	TSPOAP1	TSPO Associated Protein 1	0.92054975
1514	MTRFR	Mitochondrial Translation Release Factor In Rescue	0.917355597
1515	SLC17A6	Solute Carrier Family 17 Member 6	0.916417003
1516	VLDLR	Very Low Density Lipoprotein Receptor	0.911523819
1517	WNK4	WNK Lysine Deficient Protein Kinase 4	0.910675228
1518	DISC1	DISC1 Scaffold Protein	0.904917002
1519	HTR2B	5-Hydroxytryptamine Receptor 2B	0.904917002
1520	IL1RAPL2	Interleukin 1 Receptor Accessory Protein Like 2	0.904917002
1521	SI	Sucrase-Isomaltase	0.902408123
1522	HOPX	HOP Homeobox	0.902004302
1523	PGM3	Phosphoglucomutase 3	0.896780133
1524	FGF21	Fibroblast Growth Factor 21	0.896113336
1525	KCNMB1	Potassium Calcium-Activated Channel Subfamily M Regulatory Beta Subunit 1	0.895946205
1526	RNASE1	Ribonuclease A Family Member 1, Pancreatic	0.893325448
1527	PRKAG2	Protein Kinase AMP-Activated Non-Catalytic Subunit Gamma 2	0.889955521
1528	NIPAL4	NIPA Like Domain Containing 4	0.880901992
1529	BCL2A1	BCL2 Related Protein A1	0.878360629
1530	ARF1	ADP Ribosylation Factor 1	0.877697945
1531	NRTN	Neurturin	0.87362957
1532	THPO	Thrombopoietin	0.872451663
1533	RBP3	Retinol Binding Protein 3	0.869126439
1534	SLC9A9	Solute Carrier Family 9 Member A9	0.862014532
1535	TSLP	Thymic Stromal Lymphopoietin	0.857244253
1536	DDX39B	DExD-Box Helicase 39B	0.854114413
1537	HLA-DMA	Major Histocompatibility Complex, Class II, DM Alpha	0.854114413
1538	SH2B3	SH2B Adaptor Protein 3	0.847670197
1539	IL21R	Interleukin 21 Receptor	0.847670197
1540	PTPRD	Protein Tyrosine Phosphatase Receptor Type D	0.847067118
1541	DNMT3L	DNA Methyltransferase 3 Like	0.844476104
1542	NCOA4	Nuclear Receptor Coactivator 4	0.844476104
1543	ZFR	Zinc Finger RNA Binding Protein	0.844476104
1544	SNIP1	Smad Nuclear Interacting Protein 1	0.842150509
1545	CEP83	Centrosomal Protein 83	0.839470863

1546	ABCA4	ATP Binding Cassette Subfamily A Member 4	0.836407065
1547	EFHC1	EF-Hand Domain Containing 1	0.836407065
1548	COG2	Component Of Oligomeric Golgi Complex 2	0.836407065
1549	RARS1	Arginyl-TRNA Synthetase 1	0.830544949
1550	PTH1R	Parathyroid Hormone 1 Receptor	0.828736067
1551	GYS1	Glycogen Synthase 1	0.828736067
1552	TRAF6	TNF Receptor Associated Factor 6	0.828736067
1553	PTCHD1	Patched Domain Containing 1	0.825545728
1554	PER2	Period Circadian Regulator 2	0.823083341
1555	ZMYND11	Zinc Finger MYND-Type Containing 11	0.823083341
1556	CELF4	CUGBP Elav-Like Family Member 4	0.823083341
1557	SHANK1	SH3 And Multiple Ankyrin Repeat Domains 1	0.823083341
1558	IBSP	Integrin Binding Sialoprotein	0.81422627
1559	SALL3	Spalt Like Transcription Factor 3	0.812296629
1560	TRPC4	Transient Receptor Potential Cation Channel Subfamily C Member 4	0.811220109
1561	FGF23	Fibroblast Growth Factor 23	0.810182035
1562	NR5A2	Nuclear Receptor Subfamily 5 Group A Member 2	0.80978936
1563	TJP2	Tight Junction Protein 2	0.80978936
1564	FCGR3B	Fc Gamma Receptor IIIb	0.80402422
1565	IL2RG	Interleukin 2 Receptor Subunit Gamma	0.798371494
1566	ADRA2C	Adrenoceptor Alpha 2C	0.798371494
1567	SYN1	Synapsin I	0.798371494
1568	CNTN4	Contactin 4	0.798371494
1569	MPL	MPL Proto-Oncogene, Thrombopoietin Receptor	0.759317875
1570	ANK3	Ankyrin 3	0.758074045
1571	DLGAP2	DLG Associated Protein 2	0.758074045
1572	PDS5B	PDS5 Cohesin Associated Factor B	0.757665396
1573	MIR1908	MicroRNA 1908	0.757665396
1574	ATP2A2	ATPase Sarcoplasmic/Endoplasmic Reticulum Ca2+ Transporting 2	0.752779543
1575	TNFRSF17	TNF Receptor Superfamily Member 17	0.751077175
1576	CACNA1F	Calcium Voltage-Gated Channel Subunit Alpha1 F	0.751077175
1577	FCGR1A	Fc Gamma Receptor Ia	0.751077175
1578	KCNMB2	Potassium Calcium-Activated Channel Subfamily M Regulatory Beta Subunit 2	0.751077175
1579	SUGCT	Succinyl-CoA:Glutarate-CoA Transferase	0.742123008
1580	PYGM	Glycogen Phosphorylase, Muscle Associated	0.723858058
1581	ME2	Malic Enzyme 2	0.723858058
1582	LPA	Lipoprotein(A)	0.723858058
1583	RGS1	Regulator Of G Protein Signaling 1	0.723858058
1584	MT-ND6	Mitochondrially Encoded NADH:Ubiquinone Oxidoreductase Core Subunit 6	0.723858058

1585	BTNL2	Butyrophilin Like 2	0.71741116
1586	SOST	Sclerostin	0.715286553
1587	GRK1	G Protein-Coupled Receptor Kinase 1	0.696433842
1588	NDUFS4	NADH:Ubiquinone Oxidoreductase Subunit S4	0.695156932
1589	SLC22A1	Solute Carrier Family 22 Member 1	0.685729921
1590	HPS4	HPS4 Biogenesis Of Lysosomal Organelles Complex 3 Subunit 2	0.671705842
1591	PCSK9	Proprotein Convertase Subtilisin/Kexin Type 9	0.669243455
1592	TLR7	Toll Like Receptor 7	0.669243455
1593	ANGPTL3	Angiopoietin Like 3	0.669243455
1594	LAT	Linker For Activation Of T Cells	0.669243455
1595	CACNA1I	Calcium Voltage-Gated Channel Subunit Alpha1 I	0.669243455
1596	GATM	Glycine Amidinotransferase	0.669243455
1597	CLN3	CLN3 Lysosomal/Endosomal Transmembrane Protein, Battenin	0.669243455
1598	TMPRSS6	Transmembrane Serine Protease 6	0.669243455
1599	TNFRSF4	TNF Receptor Superfamily Member 4	0.669243455
1600	PHEX	Phosphate Regulating Endopeptidase X-Linked	0.669243455
1601	CLN8	CLN8 Transmembrane ER And ERGIC Protein	0.669243455
1602	SAR1B	Secretion Associated Ras Related GTPase 1B	0.669243455
1603	EPM2AIP1	EPM2A Interacting Protein 1	0.669243455
1604	RC3H1	Ring Finger And CCCH-Type Domains 1	0.669243455
1605	AOC1	Amine Oxidase Copper Containing 1	0.662194371
1606	CYSLTR2	Cysteinyl Leukotriene Receptor 2	0.656149268
1607	GABRD	Gamma-Aminobutyric Acid Type A Receptor Subunit Delta	0.65321064
1608	GREM2	Gremlin 2, DAN Family BMP Antagonist	0.645334423
1609	KCNH2	Potassium Voltage-Gated Channel Subfamily H Member 2	0.644531608
1610	DOCK8	Dedicator Of Cytokinesis 8	0.644531608
1611	UBE2L3	Ubiquitin Conjugating Enzyme E2 L3	0.644531608
1612	APOH	Apolipoprotein H	0.644531608
1613	LGI1	Leucine Rich Glioma Inactivated 1	0.644531608
1614	SLC22A4	Solute Carrier Family 22 Member 4	0.644531608
1615	SNRPN	Small Nuclear Ribonucleoprotein Polypeptide N	0.644531608
1616	VAPB	VAMP Associated Protein B And C	0.644531608
1617	SSB	Small RNA Binding Exonuclease Protection Factor La	0.644531608
1618	DYNC1I2	Dynein Cytoplasmic 1 Intermediate Chain 2	0.644531608
1619	MADCAM1	Mucosal Vascular Addressin Cell Adhesion Molecule 1	0.644531608

1620	ELAVL4	ELAV Like RNA Binding Protein 4	0.644531608
1621	LYST	Lysosomal Trafficking Regulator	0.644531608
1622	DIRAS3	DIRAS Family GTPase 3	0.644531608
1623	RARG	Retinoic Acid Receptor Gamma	0.642204583
1624	NFATC3	Nuclear Factor Of Activated T Cells 3	0.642204583
1625	HLA-DOB	Major Histocompatibility Complex, Class II, DO Beta	0.642204583
1626	PTPRN	Protein Tyrosine Phosphatase Receptor Type N	0.640028536
1627	KCNJ6	Potassium Inwardly Rectifying Channel Subfamily J Member 6	0.639873028
1628	PRPH	Peripherin	0.639873028
1629	ABCA12	ATP Binding Cassette Subfamily A Member 12	0.639873028
1630	CHGB	Chromogranin B	0.639873028
1631	GOSR1	Golgi SNAP Receptor Complex Member 1	0.639873028
1632	NPY5R	Neuropeptide Y Receptor Y5	0.638183951
1633	LOC126806173	BRD4-Independent Group 4 Enhancer GRCh37_chr2:27676057-27677256	0.635760427
1634	PTGER1	Prostaglandin E Receptor 1	0.626778841
1635	CHST5	Carbohydrate Sulfotransferase 5	0.624497294
1636	FADS2	Fatty Acid Desaturase 2	0.616826296
1637	KCNQ4	Potassium Voltage-Gated Channel Subfamily Q Member 4	0.600500345
1638	NISCH	Nischarin	0.578012288
1639	ACVR2B	Activin A Receptor Type 2B	0.572119951
1640	MITF	Melanocyte Inducing Transcription Factor	0.572119951
1641	HTR7	5-Hydroxytryptamine Receptor 7	0.550922811
1642	CGN	Cingulin	0.550922811
1643	CDK5R1	Cyclin Dependent Kinase 5 Regulatory Subunit 1	0.549378514
1644	CTRL	Chymotrypsin Like	0.545755625
1645	YES1	YES Proto-Oncogene 1, Src Family Tyrosine Kinase	0.544719934
1646	GUCA2A	Guanylate Cyclase Activator 2A	0.542749465
1647	STIM1	Stromal Interaction Molecule 1	0.540869772
1648	STAT2	Signal Transducer And Activator Of Transcription 2	0.540869772
1649	SEL1L3	SEL1L Family Member 3	0.540869772
1650	GFPT1	Glutamine--Fructose-6-Phosphate Transaminase 1	0.539167345
1651	MVK	Mevalonate Kinase	0.539167345
1652	FCER2	Fc Epsilon Receptor II	0.539167345
1653	CHRD	Chordin	0.539167345
1654	GUCA1A	Guanylate Cyclase Activator 1A	0.539167345
1655	KRTCAP3	Keratinocyte Associated Protein 3	0.539167345
1656	CARS1	Cysteinyl-TRNA Synthetase 1	0.536813974
1657	DEFA5	Defensin Alpha 5	0.533734679
1658	NPRL2	NPR2 Like, GATOR1 Complex Subunit	0.523397684

1659	SERPINA12	Serpin Family A Member 12	0.523397684
1660	NPRL3	NPR3 Like, GATOR1 Complex Subunit	0.523397684
1661	TFDP1	Transcription Factor Dp-1	0.496264815
1662	MYO9B	Myosin IXB	0.48618865
1663	TNFSF13	TNF Superfamily Member 13	0.48618865
1664	GALR3	Galanin Receptor 3	0.48618865
1665	LRP8	LDL Receptor Related Protein 8	0.48618865
1666	GALR2	Galanin Receptor 2	0.48618865
1667	GFRA4	GDNF Family Receptor Alpha 4	0.48618865
1668	TLX1NB	TLX1 Neighbor	0.48618865
1669	OTX2	Orthodenticle Homeobox 2	0.459796071
1670	ACSL4	Acyl-CoA Synthetase Long Chain Family Member 4	0.459796071
1671	LPIN1	Lipin 1	0.457333684
1672	LBP	Lipopolysaccharide Binding Protein	0.457333684
1673	DNAJB9	DnaJ Heat Shock Protein Family (Hsp40) Member B9	0.457333684
1674	MRGPRX2	MAS Related GPR Family Member X2	0.457333684
1675	ELOA2	Elongin A2	0.457333684
1676	KLKB1	Kallikrein B1	0.432621837
1677	TRAK1	Trafficking Kinesin Protein 1	0.432621837
1678	ADCY5	Adenylate Cyclase 5	0.427963197
1679	CHRNB1	Cholinergic Receptor Nicotinic Beta 1 Subunit	0.427963197
1680	CIC	Capicua Transcriptional Repressor	0.427963197
1681	RAPSN	Receptor Associated Protein Of The Synapse	0.427963197
1682	SLC22A2	Solute Carrier Family 22 Member 2	0.424372196
1683	LOC106099062	HBB Recombination Region	0.389065087
1684	LOC107133510	Origin Of Replication At HBB	0.389065087
1685	CACNA1B	Calcium Voltage-Gated Channel Subunit Alpha1 B	0.388590515
1686	SLC22A3	Solute Carrier Family 22 Member 3	0.387888849
1687	TBXA2R	Thromboxane A2 Receptor	0.358308196
1688	CYSLTR1	Cysteinyl Leukotriene Receptor 1	0.358308196
1689	DAO	D-Amino Acid Oxidase	0.340342849
1690	NPPC	Natriuretic Peptide C	0.340342849
1691	LOC100507346	Uncharacterized LOC100507346	0.340342849
1692	ROCR	Regulator Of Chondrogenesis RNA	0.274278849
1693	MIR3651	MicroRNA 3651	0.274278849

Disclaimer/Publisher's Note: The statements, opinions and data contained in all publications are solely those of the individual author(s) and contributor(s) and not of MDPI and/or the editor(s). MDPI and/or the editor(s) disclaim responsibility for any injury to people or property resulting from any ideas, methods, instructions or products referred to in the content.
